# Social interactions in online eating disorder communities: A network perspective

**DOI:** 10.1371/journal.pone.0200800

**Published:** 2018-07-30

**Authors:** Tao Wang, Markus Brede, Antonella Ianni, Emmanouil Mentzakis

**Affiliations:** 1 ESRC Doctoral Training Centre, University of Southampton, Southampton, United Kingdom; 2 The Alan Turing Institute, London, United Kingdom; 3 Department of Electronics and Computer Science, University of Southampton, Southampton, United Kingdom; 4 Department of Economics, University of Southampton, Southampton, United Kingdom; East China Normal University, CHINA

## Abstract

Online health communities facilitate communication among people with health problems. Most prior studies focus on examining characteristics of these communities in sharing content, while limited work has explored social interactions between communities with different stances on a health problem. Here, we analyse a large communication network of individuals affected by eating disorders on Twitter and explore how communities of individuals with different stances on the disease interact online. Based on a large set of tweets posted by individuals who self-identify with eating disorders online, we establish the existence of two communities: a large community reinforcing disordered eating behaviours and a second, smaller community supporting efforts to recover from the disease. We find that individuals tend to mainly interact with others within the same community, with limited interactions across communities and inter-community interactions characterized by more negative emotions than intra-community interactions. Moreover, by studying the associations between individuals’ behavioural characteristics and interpersonal connections in the communication network, we present the first large-scale investigation of social norms in online health communities, particularly on how a community approves of individuals’ behaviours. Our findings shed new light on how people form online health communities and can have broad clinical implications on disease prevention and online intervention.

## Introduction

Eating disorders (ED), such as anorexia nervosa and bulimia, are complex mental illnesses that can lead to serious health consequences including many intractable co-morbidities [1] and the highest mortality rate of any mental illness [2]. More than 2.7% of American 13-17 year olds [3] and 725,000 British people [4] suffer from ED, in an upward trend over time. Despite the seriousness and prevalence of this disease, it is hard to reach those who would benefit from treatment [5]. People often conceal their ED symptoms and many never seek professional help or treatment due to feelings of shame or fear of stigma [5–7]. To remain anonymous, most sufferers seek social support or disease-related information from online communities, particularly via social media sites like Twitter and Facebook [5, 8, 9]. However, not all the online communities offer healthy advice and recovery-oriented support. As explained below, some communities in fact promote harmful content and health-threatening behaviours, which has been a public health concern [10–13]. One area that is receiving increasing attention in public health research is identifying the characteristics and relationships of online communities with different stances on health problems, which has many applications in enhancing positive and reducing negative impacts of these communities, disease prevention, and online intervention [14–16].

Psychologists and clinicians have long studied online ED communities [12, 17–19]. The focus in this area has often been on pro-ED (e.g., pro-anorexia or pro-ana) communities which are featured by a stance to glorify ED (anorexia in particular) as a legitimate lifestyle choice rather than an illness [12, 13, 20]. These communities engage in disseminating content that encourages an unrealistic ideal of thinness and inspires people to lose weight, as well as tips on how to become and stay extremely thin [12, 13, 18, 19, 21]. Members of these communities display a more negative perception of body image, a higher drive for losing weight, and an increased likelihood to adopt disordered eating behaviours and maintain ED, which has become a major public health concern [9, 12, 13, 22–24]. More recently, attention has been turned from pro-ED communities to others that treat ED simply as an illness online, one typical example being so-called pro-recovery communities where members share treatment advice and provide support for people moving towards recovery [8, 25, 26]. The focus in this research has often been on the characterization and comparison of content posted by different communities online, e.g., demonstrating that pro-ED and pro-recovery individuals have distinct linguistic styles and language usages in online self-presentation [8, 26], pro-recovery content received more positive comments than pro-ED content on YouTube [11], individuals’ language use provides useful diagnostic information (e.g., emotional states and thoughts) for their severities of ED [27, 28] and indicates signs of recovery [25].

Despite providing useful insights, previous studies have several limitations. First, most previous studies focus on analysis of user-generated content online; few studies have considered social interactions among individuals. However, social networks play an important role when interpreting health-related behaviours, as our concerns, behaviours and health states are influenced by the network of people with whom we interact [29]. One pioneering study has examined interactions between 491 pro-ED and pro-recovery users via photo sharing on Flickr [10]. Yet, what dictates the interactions of individuals having different stances on ED is still under-explored. Second, a common approach for collecting data in previous studies is filtering users who post content containing a pre-defined set of keywords that relate to ED [8, 10, 11, 25]. However, a relatively small set of keywords can hardly characterize the entire community, as people can use a wide range of lexical variants to express the same content online [30–33]. Even in cases where a complete set of pattern matching rules can be obtained, people who talk about ED online may not suffer from the disease. Thus, these content-filtering based data collection methods often suffer from poor quality of data and can lead to misleading results. Finally, online ED communities studied in prior work are confined to groups of users who post certain content that researchers are interested in [8, 10, 11, 25]. This leads to a systematic exclusion of certain individuals from research. So far, the natural groupings among individuals affected by ED online remains unclear.

Here, we explore how individuals with different stances on ED interact and associate with different communities online. Studying the interactions among different communities of individuals can enhance our understanding of the affiliations of individuals in communities through the characteristics of relations between and within communities, instead of the characteristics of each community in isolation. To this end, we collect a large set of individuals who self-identified with ED in their Twitter profile descriptions using a snowball sampling method [34] and study individuals’ direct conversations through “reply” and “mention” interactions on Twitter. We focus on the Twitter platform due to its anonymous and pervasive nature, along with its very limited attempts to censor content on ED [5]. This allows us to study online ED communities in a non-reactive way.

The main contributions of this work are as follows. First, we present a clustering analysis based on users’ posting interests to explore natural groupings of users affected by ED online. Rather than assuming a priori that communities are featured by a certain posting pattern in prior studies [8, 10, 11, 25], this unsupervised approach finds communities of users based on the similarity of users’ posting interests. Second, we develop an automated approach based on sentiment analysis techniques [35] to identify the stance of an online community on a health problem like ED. Compared to previous qualitative methods [5, 18, 19, 36], this approach is more effective to handle large volumes of user-generated data online. Third, we represent users’ interactions through Twitter conversations by a directed, weighted communication network and measure the network structures to reveal how different communities of users interact with one another. Network-based representation and analysis have been shown to be an effective approach to uncover and characterize the patterns of interactions in complex systems such as human interactions [10, 29, 37] and food culture [38, 39]. Finally, we explore the underlying mechanisms that dictate users’ social interactions by studying users’ behavioural characteristics (e.g., social activities and language use online) and social norms within an online community [40]. As elaborated below, we find that users’ psychological properties reflected by their behaviours of language use in tweets can strongly shape their social interactions online and affect their positions in social networks, in different ways in communities that have different social norms. To the best of our knowledge, this is the first study of online ED communities that analyses their social interactions and norms based on a large sample of data. It provides a new perspective to understand how people form and maintain online health communities.

## Results

To analyse social interactions in online ED communities, we have gathered a large set of conversations between individuals who self-identified with ED in their Twitter profile descriptions and their Twitter friends (including followees and followers). Each Twitter conversation comprises a sequence of tweets, where each tweet is a message used by a user to reply to or mention others. In this work, we focus on studying users’ conversations around ED. By projecting these conversations onto the users who send and receive a message, we build a directed, weighted social network connecting 6,169 users with 11,056 edges. An edge *e*_*i*,*j*_ runs from a node representing user *i* to a node representing user *j* if *i* mentions or replies to *j* in a tweet, indicating that information propagates from *i* to *j*. The interaction strength of an edge *e*_*i*,*j*_ is weighted by the count of mentions and replies from user *i* to user *j*. See Methods and *Supporting Information* (*SI*) for details.

Based on this dataset, we have performed the following analyses. First, we explore natural groupings of users who engage in ED-related conversations on Twitter and identify the stances of different groups/communities of users on ED. Second, we characterize interactions of these communities by measuring structures of communication networks among users within the same community and across communities. Third, to obtain a more in-depth analysis of these interaction patterns, we measure individuals’ behavioural characteristics online. Finally, we explore the associations between individuals’ behavioural attributes and the organizational structure of a community by explicitly characterizing social norms within the community, focusing on how a community approves of individuals’ behavioural attributes [40]. Below, we present our findings in detail.

### User groupings

**Fig 1:**
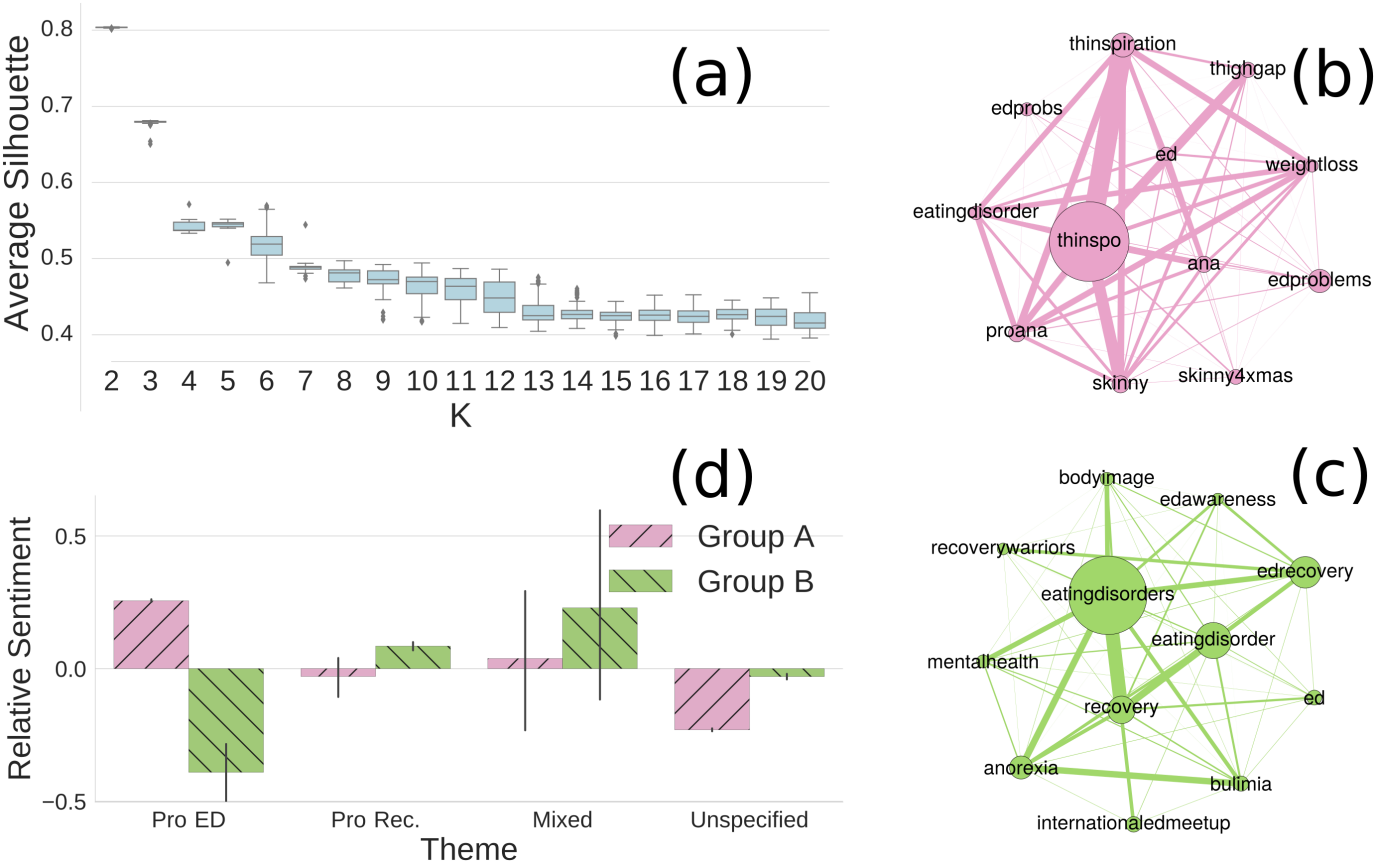
(a) Distributions of average Silhouette scores with different *k* values in *k*-means. Each box shows the quartiles of the scores obtained in 100 rounds running, and the whiskers show the rest of a distribution. (b) and (c) The most frequent hashtags and their co-occurrence networks used by two groups of users in ED-related tweets respectively. Each node is a hashtag and its size is proportional to the frequency of the tag used in a group. Edge width is proportional to the number of co-occurrences of two hashtags in tweets. (d) Average relative sentiments of two groups on different themes: *“pro-ED”* where each tweet contains a pro-ED hashtag without pro-recovery tags; *“pro-recovery”* where each tweet has a pro-recovery hashtag without pro-ED tags; *“mixed”* where a tweet has both pro-ED and pro-recovery tags; and *“unspecified”* where a tweet has neither a pro-ED nor a pro-recovery tag. Error bars denote 95% CI. Mann-Whitney *U* tests are used to assess the differences of sentiments between two groups on each theme. All *p*-values for “pro-ED”, “pro-recovery” and “unspecified” themes are *p* < 0.001, while no significant difference occurs for the “mixed” theme (see *SI*).

We profile each user by a vector that characterizes their preferences in posting content on different ED-related topics, and perform the *k*-means clustering algorithm on these vectors to find the natural groupings of users that share similar posting interests (see Methods). Fig 1(a) shows results of *k*-means with different values of *k*. The algorithm consistently produces the highest Silhouette scores [41] at *k* = 2 (with *μ* = 0.803 and *σ* = 0.001), revealing that two natural groups of users with similar characteristics are present in the sample. By inspecting content discussed in each group, we further find that these groups show two distinctive perspectives on ED. Users in group A (*n* = 5,708) focus on posting “thinspirational” content such as “#thinspo”, “#weightloss” and “#proana” (Fig 1(b)). Such content has been well-known to promote unhealthy ideals of thinness and encourage people to maintain ED as a lifestyle choice [13, 21, 42]. In contrast, users in group B (*n* = 461) often discuss mental health problems and post recovery-oriented content like “#mentalhealth” and “#edrecovery” (Fig 1(c)), indicating their intentions in promoting recovery from ED [8, 10, 25]. These results show that users involved in the ED-related discussions on Twitter can be divided into two natural groups that are likely to have a pro-ED and pro-recovery tendency respectively.

To verify whether a group indeed has pro-ED or pro-recovery stance, we measure sentiments expressed by each group of users in commenting on pro-ED and pro-recovery content (see Methods). Fig 1(d) shows the average sentiments of the two groups of users towards content on different themes, where the results are normalized based on the mean sentiment and standard deviation of a whole group expressed in all the ED-related tweets (so called *relative sentiments*, see *SI*). The two groups of users show clearly different stances on ED. Users in group A have positive comments on *“pro-ED”* content and relatively negative comments on *“pro-recovery”* content, revealing that these users typically promote negative body image and disordered eating behaviours. In contrast, users in group B have a negative view on *“pro-ED”* content and a positive view on *“pro-recovery”* content, showing that these users oppose pro-ED behaviours and encourage people to recover from ED. These results confirm that group A can be identified as a pro-ED community while group B can be identified as a pro-recovery community. To ensure the reliability of our results, we also manually annotate the presence of a pro-ED or pro-recovery tendency for a random set of users. Our annotations show very good agreement with the assignments produced by the algorithms (Cohen’s *κ* = 0.85, see *SI*).

### Network structures

**Fig 2:**
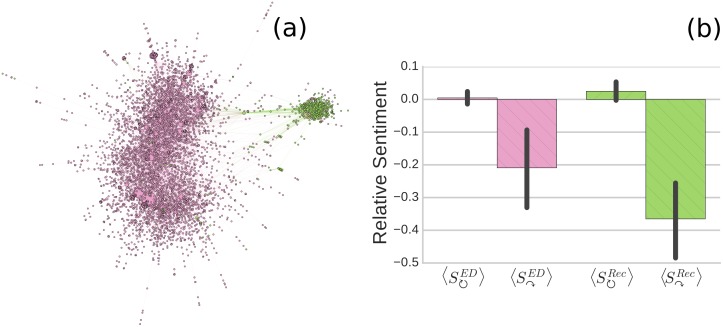
(a) The communication network of users in pro-ED and pro-recovery communities, laid out by ForceAtlas2 [44]. Each node represents a user and edges represent mentioning or replying relationships. Red nodes denote pro-ED users and blue nodes denote pro-recovery users. Node size is proportional to in-degree. (b) Average relative sentiments of intra- and inter-community messages  *S*_↻_‪ and  *S*_↷_‪ sourced from pro-ED (*ED*) and pro-recovery (*Rec*) communities respectively. Error bars denote 95% CI. Differences between *S*_↻_ and *S*_↷_ are significant (*p* < 0.01) in *U* tests in both two communities.

Based on users’ community memberships identified above and their direct communication, we visualize the communication network between pro-ED and pro-recovery communities in Fig 2(a). One clear feature shown in this figure is a division of the network into two densely connected sub-graphs, where each sub-graph consists primarily of users belonging to the same community. We measure the strength of division of the communication network into the pro-ED and pro-recovery communities (as assigned based only on users’ posting interests without considering their structural connections in the previous section) by Newman’s normalized modularity [43]. We find that the communication network is highly segregated by users’ community identities, with the normalized modularity *r* = 0.88 (*z* = 90.88, *p* ≪ 0.001 compared to a null model, see Methods). The segregated social circles are likely associated with the disagreement or conflict between these communities. We illustrate this in Fig 2(b) which compares average sentiments expressed in intra- and inter-community messages, *S*_↻_ and *S*_↷_. All results are normalized based on the mean sentiment and standard deviation of all messages sourced from a whole community (see *SI*). In both pro-ED and pro-recovery communities, inter-community interactions *S*_↷_ carry more negative emotions than intra-community interactions *S*_↻_, strongly demonstrating the disagreement between the two communities.

We next examine the network structures of pro-recovery and pro-ED communities in more detail. Table 1 shows the statistical properties of intra- and inter-community networks among pro-ED and pro-recovery users. The size of the network among pro-ED users (accounting for 93% of the whole user sample in our data) is larger than that among pro-recovery users. However, pro-recovery users have more dense connections (see 〈*k*〉), as compared to pro-ED users. The smaller value of average path length (see *L*) in the pro-recovery network implies that pro-recovery users are more closely connected with one another. While the two communities have several disconnected components (see #*Comp*.), most users (97.8% pro-ED users and 84.2% pro-recovery users) are connected in the giant components (see *GCR*). The results of reciprocity *R* and clustering coefficient *C* indicate that pro-recovery users are more likely to reciprocate the interactions they have received from others and cluster together. Both reciprocity and transitivity occur more than expected by chance in each community (see *z*_*R*_ and *z*_*C*_). Aligning with evidence on most online social networks [46], both communities show disassortative mixing by degree, i.e., high-degree nodes or hubs tend to be attached to low-degree or peripheral nodes. Compared to random networks, the pro-ED network shows stronger dissortativity than the pro-recovery network (see *z*_*A*_), indicating that the pro-ED community has a more pronounced core-periphery network organization. Due to the dominant number of pro-ED users in the user sample, the inter-community (i.e., *entire*) network show similar topological characteristics to the intra-community network of pro-ED users. These comparisons of network properties emphasize that pro-ED and pro-recovery users have different interaction patterns online and have formed communities with different organizational structures.

**Table 1:** Statistics of the communication networks among pro-ED and pro-recovery communities. Total number of nodes (*N*); number of edges (*E*); average degree per node (〈*k*〉); average shortest path length of connected node pairs (*L*); number of weakly connected components (#*Comp*.); ratio of nodes in the giant connected component (*GCR*); reciprocity measuring the likelihood of nodes with mutual links (*R*); global clustering coefficient (or transitivity) measuring the probability that two neighbours of a node are connected (*C*); assortativity coefficient of degree measuring the preference for nodes to link to others with similar degree values (*A*). Degree assortativity measured here are the correlations between source out-degree and destination in-degree [43], and *z*_*X*_ denotes the *z*-score of a property *X* observed in an empirical network compared to those observed in null models, i.e., randomized networks by preserving the degrees of the empirical network [45].

Network	*N*	*E*	〈*k*〉	*L*	#*Comp*.	*GCR*	*R*	*C*	*A*	*z*_*R*_	*z*_*C*_	*z*_*A*_
Pro-ED	5,708	9,023	1.58	10.76	114	97.8%	0.03	0.01	-0.13	90.45	0.33	-12.16
Pro-Rec.	461	1,666	3.61	3.95	62	84.2%	0.16	0.19	-0.13	20.12	10.62	-5.57
Entire	6,169	11,056	1.79	10.20	1	100.0%	0.05	0.03	-0.14	113.41	32.14	-14.45

### Behavioural characteristics

To understand users’ interaction patterns, we conduct a detailed analysis and comparison of behaviours of the pro-ED and pro-recovery users on Twitter. We focus on characterizing users’ behaviours on social activities and language use in tweets which have been well examined in previous studies [8, 10, 25]. A summary of behavioural characteristics of users in each community is reported in Table 2. We see that pro-ED and pro-recovery individuals display clearly distinctive behaviours online. Compared to pro-recovery users, pro-ED users are less active in socializing (see #followees) and generating content (see #tweets); posts of pro-ED users receive less audience (see #followers) on Twitter. Similar findings have been reported for other platforms like Tumblr [8]. The results on average activities per day show that pro-ED users are more active in following and tweeting per day, while pro-recovery users tend to attract more audience per day. Further, pro-ED users prefer to re-tweet others (see %re-tweet) and interact less with others by mentions and replies (see %mention and %reply); they tend to re-tweet content from a wider variety of people (see *H*(re-tweet)) but mention and reply to only a specific set of users (see *H*(mention) and *H*(reply)). As re-tweeting is a key part of the process of community formation and information diffusion on Twitter [47], these results show that pro-ED users use Twitter as a community engagement tool rather than a communication tool.

**Table 2:** Comparing communities in social activities and language use, where measures on language use count the percentages of words that reflect different psychometric properties, such as concerns, emotions and thinking styles, in a user’s historical tweets. Two-sided Mann-Whitney *U* tests evaluate differences between groups, significance levels with Bonferroni correction: * *p* < 0.05/*m*; ** *p* < 0.01/*m*; *** *p* < 0.001/*m* where *m* = 22.

*Measure*	*Description*	*Pro-ED (μ* ± *σ)*	*Pro-Rec.(μ* ± *σ)*	*z*	*p*
**Social Activities**					
#Followees	Number of total followees	543.70 ± 1,096.09	1,561.58 ± 4,022.53	-14.82	0.000 ***
#Tweets	Number of total tweets	2,573.03 ± 6,515.83	5,485.02 ± 10,166.14	-9.24	0.000 ***
#Followers	Number of total followers	1,339.11 ± 27,384.37	16,299.21 ± 128,844.20	-17.52	0.000 ***
#Followees/day	Average number of followees per day	2.22 ± 7.23	1.72 ± 6.18	3.36	0.001 *
#Tweets/day	Average number of tweets per day	5.48 ± 9.01	4.13 ± 8.73	7.69	0.000 ***
#Followers/day	Average number of followers per day	2.41 ± 12.36	7.51 ± 46.21	-3.81	0.000 **
%Re-tweet	Ratio of re-tweets in historical posts	0.30 ± 0.20	0.21 ± 0.18	9.76	0.000 ***
%Mention	Ratio of posts with mentions	0.31 ± 0.17	0.36 ± 0.20	-5.08	0.000 ***
%Reply	Ratio of posts with replies	0.10 ± 0.09	0.15 ± 0.13	-7.10	0.000 ***
*H*(Re-tweet)	Entropy of re-tweeting others	4.28 ± 1.20	3.36 ± 1.08	16.97	0.000 ***
*H*(Mention)	Entropy of mentioning others	2.33 ± 1.09	2.91 ± 1.04	-11.76	0.000 ***
*H*(Reply)	Entropy of replying others	2.71 ± 1.21	2.87 ± 1.05	-3.09	0.002 *
**Language Use**					
Body	Concerns of body image	0.02 ± 0.01	0.01 ± 0.01	25.68	0.000 ***
Ingest	Concerns of ingestion	0.03 ± 0.02	0.02 ± 0.02	13.41	0.000 ***
Health	Concerns of health	0.02 ± 0.01	0.02 ± 0.01	0.97	0.334
I	1st personal singular use	0.11 ± 0.03	0.04 ± 0.03	30.94	0.000 ***
We	1st personal plural use	0.00 ± 0.00	0.01 ± 0.01	-25.52	0.000 ***
Social	Social concerns	0.08 ± 0.02	0.11 ± 0.03	-19.78	0.000 ***
Swear	Abusive language	0.01 ± 0.01	0.00 ± 0.00	28.84	0.000 ***
Negate	Negation use	0.03 ± 0.01	0.02 ± 0.01	25.86	0.000 ***
Posemo	Positive emotions	0.05 ± 0.01	0.06 ± 0.02	-18.34	0.000 ***
Negemo	Negative emotions	0.04 ± 0.01	0.02 ± 0.01	26.14	0.000 ***

From the psychometric properties reflected by users’ language use in tweets, we find that pro-ED users are more concerned about body image (see *body* in Table 2) and ingestion (see *ingest*), which is an important signal of ED [48]. Also, pro-ED users typically use the 1st person singular (see *I*), reflecting their loneliness, self-focused attention and psychological distancing from others [49]. In contrast, pro-recovery users often use the 1st person plural (see *we*), showing their social embedding within the group. These results are confirmed by that pro-ED users have less social concerns (see *social*). This can be due to feelings of social isolation and rejection, or due to the lack of social support for those suffering from mental illness [8, 26]. Further, pro-ED users use more swear (see *swear*) and negation words (see *negate*) in their discourse on Twitter, reflecting their aggression and refusal/contradiction [50]. Pro-ED users also manifest less positive emotions (see *posemo*) but more negative emotions (e.g., sadness, anxiety and anger, see *negemo*), indicating their tendencies for depression, mental instability and irritability. The typically negative tone of pro-ED users also reflects a lowered sense of self-esteem, likely due to normative dissatisfaction with one’s body weight and shape [27]. Moreover, these results hint that users’ psychological properties are likely to shape their social networks online, e.g., less social concern and more refusal of others among pro-ED users may explain their fewer interconnections, less likelihood to cluster together and a lower reciprocity in the communication network (see Table 1).

### Community norms

Next, we present a more systematic exploration of the associations between individuals’ behavioural characteristics and the collective network structure of a community. We establish the links between individual characteristics and organizational structures from a sociological perspective and situate our analysis in the context of social norms, i.e., how a group approves of individuals’ behavioural attributes. According to the classic definition of social norms in psychological studies [40], we assume that social norms have two dimensions: (i) how much an attribute of an individual is exhibited, and (ii) how much the group approves of that attribute. We focus on users’ psychological attributes (e.g., concerns and emotions) reflected by their behaviours in language use, as these attributes are more related to psychometric indexes of ED than others [51]. We measure the amount of an attribute exhibited by the percentage of words related to the attribute in a user’s tweets (i.e., in the same way as measured in Table 2) and measure the amount of group acceptance by the user’s PageRank centrality [52] in an intra-community network. PageRank centrality quantifies how focal or popular an individual is in a network by considering all connections in the network; people who receive a greater amount of attention (e.g., in-links) have a higher centrality. In this light, the centrality metric can effectively capture the structural properties of a network, but can also be interpreted as a good measure of acceptance of an individual in a group. Then, we use the classic return potential model (RPM) [40] to explain social norms, and build regression models which use the amount of an attribute exhibited to predict the amount of group acceptance to evaluate the strength of a norm (see Methods).

**Fig 3:**
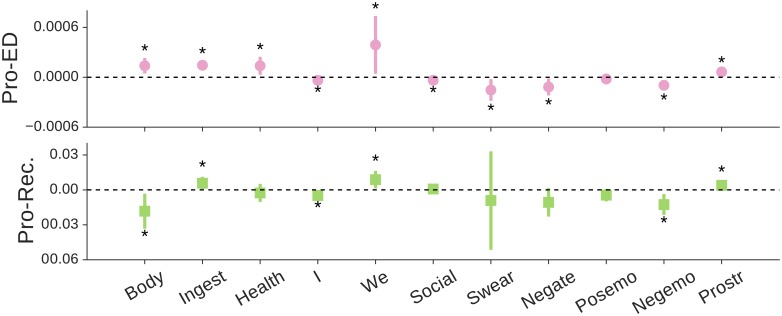
Parameters estimates *β* and 95% confidence intervals for effects of an attribute on PageRank centralities in pro-ED and pro-recovery communities, estimated using robust linear models with controls on social capital covariates (see Methods). Coefficients at significance level *p* < 0.05 are labelled with an asterisk. (*Prostr*) is the strength that a user promotes a pro-ED or pro-recovery tendency, measured by the average sentiment of the user on pro-ED or pro-recovery content in tweets (see *SI*).

Fig 3 shows estimated correlations between psychological attributes and network centralities of individuals in different communities. We find that users with more concerns about body image tend to be located more centrally in the pro-ED community. In contrast, users with more concerns about body image tend to be more peripheral in the pro-recovery community. Users who talk more about ingestion tend to be more central in both two communities. Interestingly, pro-ED users who share more information on medication and health-related materials tend to be more focal (see *health* in Fig 3); a cause may be that pro-ED individuals often share/seek advice on using medications (e.g., diuretics, enemas and laxatives) to lose weight or inhibit appetite in online communities [1]. Consistent with studies in social psychology [53], people who exhibit less self-focused attention (using less *I* and more *we*) are more popular in a social community. Also, people with more negative emotions tend to be located in the periphery of their communities. This finding aligns with previous findings in offline social networks that happy people are likely to be located in the centre of their local social networks [29], and also confirms the positive role of optimism in social network development [54]. Finally, users who show a stronger pro-ED or pro-recovery tendency tend to be more popular in the corresponding communities, emphasizing their roles as opinion leaders [15, 55].

## Discussion

In this paper, we have explored ED-related communities on Twitter and their interactions via Twitter conversations. We have shown that participants in ED-related conversations on Twitter can be divided into two main communities: a pro-ED community which promotes disordered eating behaviours; and a pro-recovery community which encourages people to recover from the disease. Consistent with prior studies of these communities on other platforms like Flickr and YouTube [10, 11], we find that people tend to interact almost exclusively with others in the same community, with extremely limited interactions between communities on Twitter. That is, people sharing similar interests and stances on ED tend to be connected within the communication network on Twitter, expressed by the presence of strong homophily [56]. This is of particular importance in reaching larger populations affected by ED through online social networks. Beyond that, our findings shed new light on the role of emotional interactions in the segregation between the two communities in social networks, i.e., more negative emotions in inter-community interactions can intensify the split in affiliation between different communities [57, 58], whereas more positive emotions in intra-community interactions can enforce social ties and strengthen pre-existing identities of members within the same community [11, 59].

We find that users in the two communities display distinctive social behaviours and psychological properties on Twitter. Compared to pro-recovery users, pro-ED users exhibit an excessive focus on body image and food ingestion, increased feelings of social isolation and self-occupation, heightened aggression and refusal, more negative emotions and less positive emotions, showing greater risk of ED and poorer mental health. These results are compatible with prior evidence that pro-ED communities exacerbate risk of ED [12, 13] through an unrealistically thin ideal [9, 23], reinforcement of an ED identity [19, 36], or exposing and adopting harmful weight loss practices [12, 13]. Also, our results show that the negative impact of pro-ED communities tends to self-reinforce through very active Twitter engagement (e.g., actively following, tweeting and re-tweeting behaviours). Similar findings that pro-ED groups are more active than pro-recovery groups have been reported for other platforms like Facebook [60].

We further find that individuals’ psychological characteristics can shape their social networks on Twitter. Characteristics that benefit community development (e.g., less self-focused attention and lowered negative tones) and behaviours that strongly indicate a community identity (e.g., actively sharing content on body image and making positive comments on pro-ED or pro-recovery content) tend to attract more attention and help actors to be more central in a social network. While our data do not allow us to identify the actual causal mechanisms of network dynamics, our results provide new insights into how people maintain order in these online communities. Our findings also indicate that central individuals in a social community are likely to act as opinion leaders in the community [55, 61]. These individuals actively promote information on a specific lifestyle (e.g., pro-ED or pro-recovery) and their central positions can further make them a credible, easily-assessable source of information. In this light, these central individuals can be more influential than others to shape health-related opinions in a community.

Our findings have relevance for public health. First, social media are not only a valuable medium for reaching individuals who are affected by ED [34], but also for identifying larger groups who seek recovery from ED and would benefit more from treatment. Second, automated analysis on social media data can complement self-report based psychiatric assessments on ED and help to tailor specific interventions for pro-ED and pro-recovery individuals through non-reactive and non-intrusive measurements of their behaviours online. Third, while online support groups have been increasingly used for promoting health behaviour change [14], here we find that the influence of these groups may be limited due to the network organization. A strong segregation between groups in social networks might undermine behavioural contagion across groups [62]. Thus, health interventions over support groups may need to account for the fact that structures and dynamics of individuals’ social networks can affect the intervention outcomes. Finally, as health promotion programs become more community oriented, community opinion leaders have been widely used in public health to promote organizational well-being [15, 16]. Traditional methods for identifying effective opinion leaders primarily rely on surveys and interviews [15]. However, these methods are often time-consuming and hard to implement in large communities. The observations from our study complement previous work on opinion-leader identification through analysing naturally occurring data on social media.

Our study has its limitations. First, this study is limited to ED communities and their communication networks on Twitter; the findings thus cannot be generalized to other communities that may function differently depending on various user-interaction habits. Second, our data is collected via Twitter APIs; we have little data on other interactions like viewing behaviours. Hence, for instance, users who actively browse content but never post any tweets, mentions, or replies are excluded from our data. Third, while our computational and manual validations show that users are likely to be correctly classified into their corresponding community (high precision), our analyses do not guarantee high recall—we missed populations that were not identified by our data collection methods. For example, our results show that the number of pro-ED users is larger than that of pro-recovery users, which aligns with prior evidence that pro-ED communities are more common than pro-recovery communities on social media [8, 10, 25]. However, this may be caused by the fact that pro-recovery users have a broader range of posting interests (not limited to ED-related topics) while pro-ED users strongly focus on sharing “thinspiration” content [10]. Thus, our data collection methods are likely to miss pro-recovery/recovered users who did not post any ED-related content in their recent tweets. Finally, all users’ health states are measured from their behaviours online; we do not have any clinical indications on their actual states. Ethical concerns and privacy issues make it unlikely to obtain such ground-truth data.

Future work will focus on exploring effective intervention strategies. We envision a system that could apply an intervention tailored to individuals’ personalized traits such as a pro-ED or pro-recovery tendency, and a core or periphery position in local social networks. For example, we can deliver warning messages or ban content for core pro-ED users; expose healthy and recovery-oriented content to periphery pro-ED users; facilitate access to social support (e.g., recommending recovery tips, professionals or other peers in recovery) for periphery pro-recovery users; and recruit or support core pro-recovery users as behaviour-change agents. Another interesting direction is to study the evolution of social interactions in online ED communities over time, so as to improve our understanding of dynamic processes in these communities. It is also important to examine the causal influence of exposure to pro-ED or pro-recovery content on health, the causal relations between behaviours and social statuses in online ED communities. Further, we will examine whether our findings are applicable to other online communities based on a different type of social media (e.g., Facebook and Instagram) or multimedia content (e.g., images and videos), and a broader range of public health problems.

## Methods

Our study protocol was approved by the Ethics Committee at the University of Southampton. All data we collected is *public* information on Twitter and available via the Twitter APIs. Any data that has been set as *private* is excluded from our study.

### Data collection

We collect a set of users who have self-identified with ED in their Twitter profile descriptions and their Twitter friends (*n* = 208,065) using a snowball sampling approach [34]. For each user, we collect up to 3,200 (the limit returned from Twitter APIs) historical tweets, resulting in a corpus of tweets (*n* = 241,243,043) in March 2016. From this corpus, we extract 633,492 ED-related tweets posted by 41,456 unique users by checking the occurrences of ED-related hashtags (e.g., “#thinspo” and “#edproblems”) in tweets. The ED-related hashtags we used are obtained by: (i) applying Infomap [63], an established method for community detection, to the co-occurrence networks of hashtags posted by self-identified ED users, resulting in topic clusters of semantically related hashtags; (ii) selecting ED-related topics based on prior evidence of ED-related content on social media [8, 21, 25]; (iii) removing generic hashtags (e.g., “#skinny” and “#food”) from the selected topics.

Based on users’ mentioning and replying relationships in these ED-related tweets, we build a communication network comprising 13,139 non-isolated nodes and 21,761 edges to represent users’ interactions in ED-related conversations. All mentions in re-tweets are excluded, as these mentions are used by the original author of a re-tweet, not by the users who re-tweeted this tweet. To filter out noise, e.g., users who occasionally mention ED, we exclude users who have less than three distinct ED-related tweets. The resulting network contains 6,775 nodes and 11,405 edges, where the largest weakly connected component has 6,169 nodes and 11,056 edges, with 7 nodes in the second-largest component. We focus on analysing the largest component due to its dominance (see *SI*, Sect. 1).

### User profiling and clustering

We profile each user by their interests in posting different ED-related hashtags, as the social signal of posting specific tags on social media has been shown to strongly indicate the tendency of an individual for a healthy or unhealthy lifestyle [5, 8, 10, 11, 25, 64]. Since multiple duplicate hashtags can represent the same event, theme or object, we shift attention from single tags, as widely used in prior work [8, 10, 25, 32], to more general categories, i.e., topics of semantically related tags. We identify the topics of hashtags by constructing a co-occurrence network of hashtags in the ED-related tweets, and detecting dense clusters in the network using the Infomap algorithm. Then, we track the sequence of hashtags that a user used in the ED-related tweets, and profile the user by a vector that consists of proportions of usage of these hashtags across the topics found above. Finally, we apply the *k*-means clustering algorithm on these vectors to group users who have similar posting interests into the same community. To identify the natural number of communities in data, we run *k*-means with different values of *k* and select the value of *k* that maximizes the average Silhouette coefficient over all samples [41]. To ensure the robustness of the results, we repeat these analyses 100 times with *k* ∈ [2, 20] and observe high consistency in the results (see *SI*, Sect. 2).

### Sentiment analysis

To examine users’ attitudes to pro-ED and pro-recovery content, we measure their sentiments expressed in pro-ED and pro-recovery tweets. We categorize pro-ED and pro-recovery tweets based on the occurrence of a pro-ED or pro-recovery hashtag in a tweet. The pro-ED and pro-recovery hashtags we used are obtained by (i) identifying pro-ED and pro-recovery topics from the topics of hashtags found in the ED-related tweets, based on previous studies on the language use in online pro-ED and pro-recovery communities [8, 10, 11, 25]; (ii) removing generic hashtags such as “#ana” and “#ed” (*SI*, Sect. 3).

SentiStrength [35] is used to measure sentiments as: (i) it is designed for short informal texts with abbreviations and slang, and thus suitable to process tweets; (ii) it accounts for linguistic rules of negations, amplifications, booster words, emoticons, spelling corrections, showing good performance in sentiment analysis [35, 65]. This tool assigns two values to each tweet: *S*_*p*_ which measures positive sentiment, ranging from 1 (not positive) to 5 (extremely positive), and *S*_*n*_ which measures negative sentiment, ranging from -1 (not negative) to -5 (extremely negative). Due to the paucity of information conveyed in short texts (up to 140 characters in tweets), previous studies suggest that measuring the overall sentiment is more accurate than measuring the two dimensions of sentiment separately [35, 65]. Following this research, we capture the sentiment polarity of each tweet with one single measure, i.e., *S* = *S*_*p*_ + *S*_*n*_, in the range of [−4,4] where 0 indicates a neutral opinion. All hashtags, URLs, re-tweet and mention marks are removed before sentiment analysis. The same pre-processing is used in measuring the sentiments of tweets that are associated with intra- and inter-community interactions (see *SI*, Sects. 3 and 4).

### Null model

We use a null model [45] to evaluate the normalized modularity (or assortativity coefficient) [43] of the communication network by users’ community labels that are assigned by the clustering algorithm based on users’ posting interests. We randomly shuffle users’ community labels and re-measure assortativity by the shuffled labels. Repeating this procedure 3,000 times, we obtain an empirical distribution of assortativity by users’ community labels, with the mean value of assortativity coefficients *μ* = 0 and the standard deviation *σ* = 0.01. Using this distribution as a baseline, we measure the deviation of the actual assortativity *A* from randomness via a *z*-score: *z* = (*A* − *μ*)/*σ*. The result is *z* = 90.88, showing that the actual value of assortativity is larger than the random values of assortativity, significantly at *p* ≪ 0.001 in a two-tailed test.

### Characterizing language use

We adopt the psycholinguistic lexicon LIWC [50] to characterize content and language use in tweets. This tool reads a given text and counts the percentages of words that reflect different emotions, thinking styles, and social concerns; it has been widely used to capture people’s psychological and health states from the words they used [8, 25, 32]. For a more reliable evaluation, we combine all historical tweets of each user as a document. All re-tweets are excluded, since they reflect cognitive attributes of their original authors rather than those of re-tweeters. After removing mention marks, hashtags and URLs, each document is split into tokens by white-space characters. Only documents containing more than 50 tokens are processed with LIWC for more trustworthy results (see *SI*, Sect. 5).

### Characterizing social norms

We measure the two dimensions of social norms by (i) the amounts of language reflecting different psychological attributes (e.g., concerns and emotions) in a user’s tweets and (ii) the centrality of the user in the social network within a community. We measure the PageRank centrality [52] due to its several advantages over other centralities (e.g., degree and eigenvector centrality): (i) it accounts for the centralities of a node’s neighbours, and (ii) it is insensitive to spammers with a large number of out-links. Due to the dominance of the giant weakly connected component in the intra-community networks and incomparable PageRank values of nodes across disconnected components, we focus on users within the giant components in the analysis of social norms. For validation, we perform the same analyses using other centrality metrics for directed, weighted networks—hubs and authorities [66]. The results are similar (see *SI*, Sect. 6.1).

To explain social norms from a more theoretical respective, a common method is the RPM, which plots the change of the amount of group acceptance with the amount of an attribute exhibited [40]. However, the RPM is primarily a descriptive model; it can hardly quantify the strength of a relation between two dimensions of social norms. Here, we follow the framework of RPM and build linear regression models to quantify these relations. Each model predicts a user’s centrality in a network based on an attribute of the user (such as concern on body or positive emotions) and covariates including the numbers of followers, tweets, followers that the user has, the fractions of tweets mentioning and replying to others, and the number of the historical tweets that the user has in our data. Given the long tailed distributions of centrality values, we use robust linear regression models, which are less sensitive to outliers or influential observations [67], to achieve robust estimations on the relations between individuals’ psychological attributes and centralities in social networks (see *SI*, Sect. 6.2).

## Supporting information

S1 Appendix (PDF)
