# Supplementary material for: Social interactions in online eating disorder communities: A network perspective
Source: PLoS One. 2018 Jul 30;13(7):e0200800. doi: 10.1371/journal.pone.0200800 (PMC6066201; doi:10.1371/journal.pone.0200800)
Supplement: S1 Appendix — (PDF) [file pone.0200800.s001.pdf]

Supporting Information
Social Interactions in Online Eating Disorder Communities: A Network Perspective

Tao Wang, Markus Brede, Antonella Ianni, and Emmanouil Mentzakis

Contents

1 Data Details **2**
- 1.1 Sampling Disordered Users . . . 2
- 1.2 Filtering ED-related Tweets . . . 3
- 1.3 Constructing Communication Network . . . 3

2 User Clustering Analysis **4**
- 2.1 Hashtag Clustering . . . 4
- 2.2 User Profiling . . . 5
- 2.3 User Clustering . . . 6

3 Community Identification **7**
- 3.1 Posting Interests . . . 7
- 3.2 Attitudes on ED-related Content . . . 7
- 3.3 Manual Annotation . . . 8

4 Emotional Interactions **9**

5 Measures on User Characteristics **10**
- 5.1 Social Activities . . . 10
- 5.2 Language Use . . . 10

6 Community Norms **10**
- 6.1 Characterizing Social Norms . . . 10
- 6.2 Regression Models . . . 11

List of Figures

- S1 Diagram of analysis . . . 2
- S2 Topic clusters in core ED users' tweets . . . 4
- S3 Degree distributions of communication networks . . . 5
- S4 Topic clusters in ED-related tweets . . . 6
- S5 Statistics of users and tweets related to ED-related themes . . . 8
- S6 Statistics of users and links in intra- and inter-community interactions . . . 9
- S7 Distributions of centralities . . . 11

List of Tables

S1 Prominent hashtags in two clusters of users 7
S2 Most frequent pro-ED and pro-recovery hashtags. 8
S3 Sentiments of users on themes of content 8
S4 Sentiments of users' interaction messages 9
S5 Centrality as a function of *body* and covariates 12
S6 Centrality as a function of *ingest* and covariates 13
S7 Centrality as a function of *health* and covariates 14
S8 Centrality as a function of *i* and covariates 15
S9 Centrality as a function of *we* and covariates 16
S10 Centrality as a function of *social* and covariates 17
S11 Centrality as a function of *swear* and covariates 18
S12 Centrality as a function of *negate* and covariates 19
S13 Centrality as a function of *posemo* and covariates 20
S14 Centrality as a function of *negemo* and covariates 21
S15 Centrality as a function of *prostrr* and covariates 22

1 Data Details

We analyse a dataset collected from Twitter, a social media platform that allows millions of users to post and interact with short messages ("tweets"). Users can "follow" others to receive their updates, forward ("re-tweet" and "RT") tweets to their own followers, or mention and reply to ("@") others in tweets. People can also label tweets with hashtags ("#") to makes it easier for users to find tweets with a specific theme or topic. All data used in our analysis is public information, available via the Twitter APIs. As shown in Fig. S1, we build our dataset in the following phases.

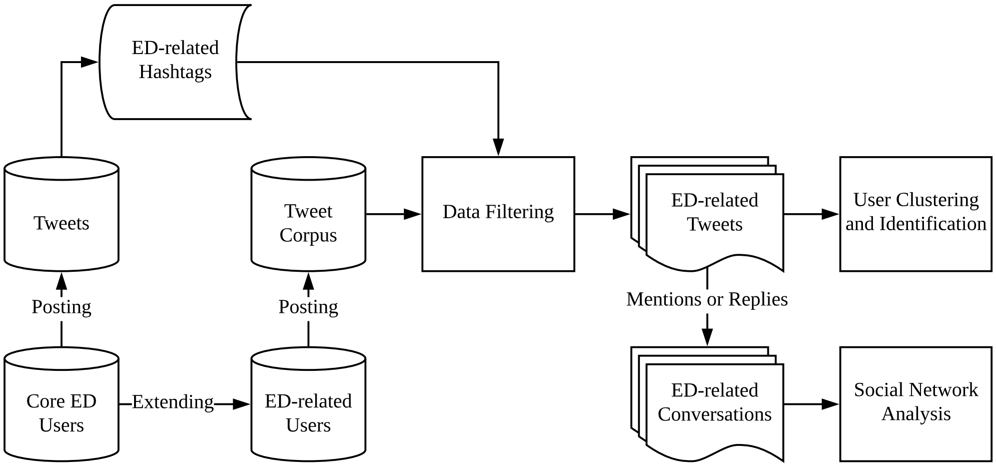

Figure S1: Diagram representing the data flow and analysis process in our study.

1.1 Sampling Disordered Users

To sample individuals with eating disorders (ED) on Twitter, we adopt an approach used in previous work for detecting ED-related communities from social media sites like Twitter [1]. We

begin by tracking the public tweet stream using "eating disorder", "anorexia", "bulimia" and "EDNOS" from Jan. 8 to 15, 2016, leading to 1,169 tweets that mention common ED. From the authors of these tweets, we obtain 33 seed users who self-diagnosed with ED. We identify users as ED-diagnosed if they self-report both ED-diagnosis information (e.g., "eating disorder", "edprob" and "proana") and personal bio-information (e.g., body weight) in their Twitter profile descriptions (i.e., a sequence of user-generated text describing their accounts below profile images). Then, we expand the set of seed users by using a snowball sampling through users' social networks of followees/followers on Twitter. At each sampling stage, we filter out non-English speaking accounts and finally obtain 3,380 unique users (called *core ED users*). Our annotation results on randomly selected 1,000 users from the core ED sample show that almost all of the checked users are suspected of having ED and 95.2% of the users are labelled as being highly likely to have ED (see [1] for more details on data collection and validation). We further collect all followees and followers of these core ED users, leading to a large sample of ED-related users ($n = 208,065$). For each user, we retrieve up to 3,200 (the limit returned from Twitter APIs) of their most recent tweets, resulting in a corpus of 241,243,043 tweets. The retrieval process finished on Mar. 2, 2016.

1.2 Filtering ED-related Tweets

To examine users' conversations on ED, we extract ED-related messages from our tweet corpus by searching for tweets that contain at least one ED-related hashtag. To identify an appropriate set of ED-related hashtags and avoid introducing bias, we detect ED-related topics from hashtags used by the core ED users. We consider hashtags posted only by the core ED users rather than those posted by the whole user sample, since the whole sample contains a large number of users who have only weak connections to ED (e.g., celebrities and marketing accounts). Not all topics collected based on hashtags used by the whole user sample are directly related to ED, thus making it harder to identify ED-related topics.

Based on the topic locality assumption that semantically similar hashtags tend to appear in the same tweets together, and hence similar hashtags are likely to be densely connected in their co-occurrence networks [2, 3], we construct an undirected, weighted hashtag co-occurrence network. In this network, each edge runs between two nodes representing two hashtags if the two hashtags co-occur in a tweet posted by the core ED users, with a weight counting the number of tweets containing the two attached hashtags. To filter out noise, we only consider hashtags used by more than three distinct users and observed in more than three tweets. The resulting network contains 4,915 nodes and 34,121 edges. Then, we detect densely connected clusters in this network by using the Infomap algorithm [4] which finds cluster structures by minimizing the description length of a random walker's movements on a network. We obtain 1,200 clusters, where 872 topic clusters have only a single hashtag and 328 clusters have more than one hashtags. Fig. S2 shows the largest topic clusters in the hashtag co-occurrence network. From these generated topic clusters, we select ED-related topics (e.g., "ED") based on previous studies on ED-related content on social media [5, 6, 7]. After removing generic tags such as "#skinny" and "#staystrong", we obtain 375 unique ED-related hashtags such as "#thinspo", "#edproblems" and "#proana". Finally, we search for tweets containing any of these tags in our tweet corpus, yielding 633,492 public ED-related tweet messages posted by 41,456 unique users.

1.3 Constructing Communication Network

We track user-user conversations/communication on ED by searching for the ED-related tweets in which authors mention or reply to other users. Since a user can join a Twitter conversation by either mentioning or replying to others in a tweet [1], we do not distinguish the two types of

[1] https://help.twitter.com/en/using-twitter/mentions-and-replies

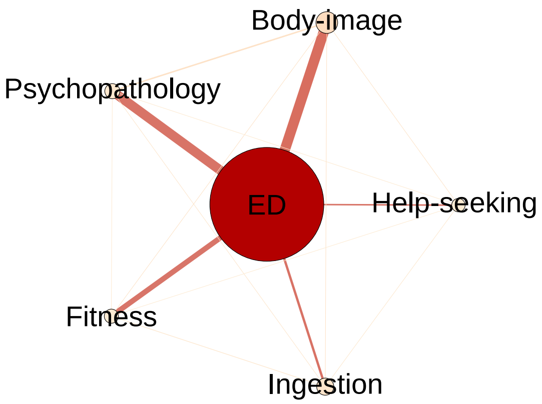

Topic	*Example #Hashtags*
ED	thinspo, edproblems, thinspiration, proana, ana, skinny, staystrong, thighgap, edprobs, ed, eatingdisorder.
Body-image	picslip, fat, fml, failure, fatass, progress, fuck, ugh, reversethinspo, fatty, ugly, gross, ew, disgusting, fail.
Ingestion	myfitnesspal, tweetwhatyoueat, twye, eatclean, healthy, yum, vegan, calories, breakfast, food, yummy.
Psychopathology	selfharm, depression, depressed, anxiety, sad, suicide, cutting, triggering, self-harmproblems, suicidal.
Fitness	fitfam, fitness, workout, getchallenged, exercise, fitfeb, noexcuses, fit, health, gym, fitfamlove, skinnyteams.
Help-seeking	replytweet, help, please, confused, curious, anafam, advice, previoustweet, lasttweet, now, prettyplease.

Figure S2: Top plot shows the largest topic clusters in the co-occurrence network of hashtags posted by the core ED users. Each node is a cluster of hashtags on the topic as labelled. Node size is proportional to the total frequency of all hashtags in the cluster, and edge width is proportional to the co-occurrences between tags from two attached clusters. Bottom table lists example hashtags of clusters, ranked by their frequencies.

interactions in this analysis. Compared with other types of interactions such as who-follows-whom and who-retweets-whom relationships on Twitter, the direct interactions through Twitter conversations have been shown to exhibit more similar characteristics to real person to person social interactions [8, 9]. Based on the reciprocal mentioning and replying relationships between users in these conversations on ED, we build a directed, weighted communication network to describe how users interact with one another. Bidirectional edges denote mutual interactions, with larger weights indicating more frequent or persistent interactions between two individuals. We only consider the interactions that both senders and recipients exist in our data. All of our analyses focus on the users connected in the communication network. Fig. S3 shows the degree distributions of communication networks among pro-ED and pro-recovery communities.

2 User Clustering Analysis

2.1 Hashtag Clustering

We characterize users' interests in ED based on their usages of hashtags in the ED-related tweets. However, multiple hashtags can be developed to represent the same event, theme or object on Twitter, e.g., both "#thinspo" and "#proana" refer to the promotion of behaviours related to anorexia nervosa and encouraging people to lose weight. To capture the semantic relatedness

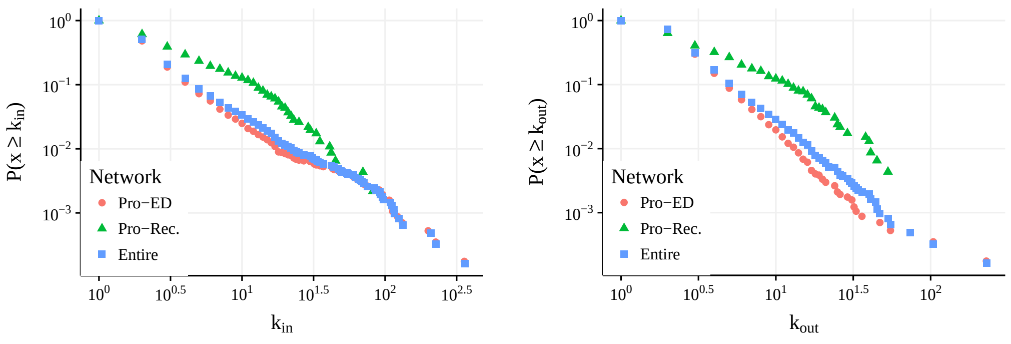

(a) (b)

Figure S3: (a) In-degree and (b) out-degree distributions of communication networks. We add 1 to all values of degrees to account for nodes with zero-degrees.

of hashtags, we shift attention from single hashtags to more general categories, i.e., clusters of semantically related hashtags. Similar to our previous method on identifying ED-related hashtags, we construct hashtag co-occurrence network from the ED-related tweets and use Infomap to detect finer-grained sub-topics on ED. After removing hashtags with low frequencies (occurring in more than 3 tweets) and engagement (used by more than 3 distinct users), the resulting network contains 5,732 nodes and 126,635 edges. We detect 140 topic clusters from the network. Fig. S4 shows the most frequent topic clusters in the hashtag co-occurrence network of the ED-related tweets.

Note that, although topics in Fig. S4 can be regarded as the sub-concepts of the "ED" topic in Fig. S2, the network in Fig. S4 is not a subgraph of the network in Fig. S2. This is because these networks are built based on tweets posted by two different sets of users, where the network in Fig. S2 is built from all tweets posted by the core ED users, while the network in Fig. S4 is built from ED-related tweets posted by the whole user sample (see Fig. S1). Moreover, an ED-related tweet can contain both an ED-related hashtag identified in Fig. S2 and other hashtags. Thus, the size of hashtag co-occurrence network in Fig. S4 is not necessary smaller than that in Fig. S2.

2.2 User Profiling

We profile users by their interests in these ED-related sub-topics found above. Given a user u, we track the sequence of n_u hashtags (with repetition) that she/he used in the ED-related tweets, $(h_1, h_2, ..., h_{n_u})$. Each hashtag h_i is attached to a topic $T(h_i)$ as:

$$T(h_i) = \begin{cases} C(h_i) & \text{if } h_i \text{ exists in the hashtag co-occurrence network} \\ T_{|C|+1} & \text{otherwise} \end{cases} \tag{1}$$

where $C(h)$ is a sub-topic containing h and $|C|$ is the number of clusters found in the hashtag co-occurrence network. $T_{|C|+1}$ is a dummy topic to host all hashtags that are not in the co-occurrence network (e.g., low-frequency tags). Each user is represented as a vector which is constructed by computing the proportions of hashtags across different sub-topics:

$$\vec{u} = \Big(P(T_1), P(T_2), ..., P(T_{|C|+1})\Big) \tag{2}$$

where

$$P(T_j) = \frac{\sum_{1 \leqslant i \leqslant n_u} I(h_i, T_j)}{n_u}, \tag{3}$$

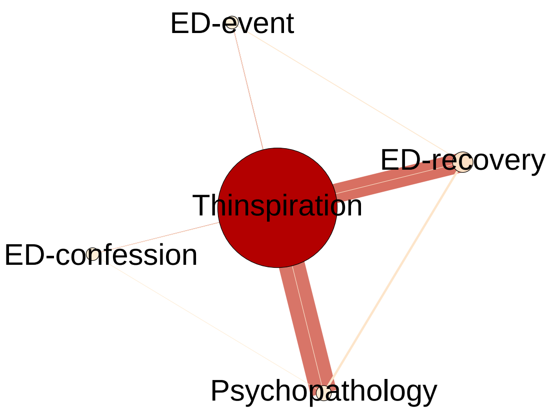

Cluster	*Example #Hashtags*
Thinspiration	thinspo, thinspiration, edproblems, skinny, ana, proana, eatingdisorder, thighgap, anorexia, ed, edprobs.
ED-recovery	eatingdisorders, edrecovery, recovery, mentalhealth, bodyimage, recoverywarriors, edawareness.
Psychopathology	depression, selfharm, depressed, anxiety, suicide, suicidal, sad, quotes, cutting, alone, cut, cat, broken.
ED-confession	bulimicprobz, anorexicprobz, edprobz, awkward, willbeskinny, selfharmprobz, wasted, noselfcontrol, noforreal.
ED-event	internationaledmeetup, edsoldiers, australia, melbourne, australianeatingdisorders, aussie, pink, pinkribbon.

Figure S4: Top plot shows the largest topic clusters in the hashtag co-occurrence network of the ED-related tweets. Each node is a cluster of hashtags on the topic as labelled. Node size is proportional to the total frequency of all hashtags in the cluster, and edge width is proportional to the co-occurrences between between tags from two attached clusters. Bottom table lists example hashtags of clusters, ranked by their frequencies.

and $I(h_i, T_j)$ is a function indicating whether hashtag h_i is associated with topic T_j, defined as:

$$I(h_i, T_j) = \begin{cases} 1 & \text{if } T(h_i) = T_j \\ 0 & \text{otherwise.} \end{cases} \quad (4)$$

2.3 User Clustering

We perform the k-means clustering algorithm on these vectors to partition users into k clusters. To identify an appropriate number of clusters in the data, we run k-means by setting different values of k (a parameter specifying the number of expected clusters) and select the value of k that maximizes the average Silhouette coefficient [10]. The Silhouette coefficient is a measure of how appropriately the data have been clustered by computing how similar an object is to its own cluster compared to other clusters. Given a set of samples $\{x_1, x_2, ..., x_n\}$, the average Silhouette score over all samples is:

$$s = \frac{1}{n} \sum_i \frac{b_i - a_i}{\max\{b_i, a_i\}}, \quad (5)$$

where a_i is the mean distance between a sample x_i and all other samples in the same cluster. b_i is the mean distance between x_i and all other samples in the next nearest cluster. We use the Euclidean distance to measure the distance between two samples. The Silhouette score ranges from -1 to 1, where a high value indicates a better clustering and scores in the range between 0.71 to 1.0 indicate a strong structure in data [11]. The value of k that maximizes the Silhouette score is

often regarded as the natural number of clusters in data. To obtain a reliable estimation on the cluster number, we run the pipeline of hashtag clustering, user profiling and clustering 100 times. In each run, we calculate the average Silhouette scores given different values of $k \in [2, 20]$.

3 Community Identification

To investigate the identities for the two groups of users found above, we (i) examine users' posting interests in the ED-related tweets, (ii) measure users' attitudes on different types of ED-related content, and (iii) manually check a random sample of users in the two groups.

3.1 Posting Interests

Table S1: The most prominent hashtags used by two groups of users, ranked by NPMI.

Group	*#Hashtags*
A	thinspo, thinspiration, edproblems, skinny, proana, thighgap, skinny4xmas, ana, edprobs, anasisters, weightloss, proed, thin, hipbones, fitspo, diet, bonespo, mia, ribs, anafamily, thinkthin, perfection, legs, edgirlprobs, collarbones, edlogic, bones, staystrong, legspo, picslip, skinny4xmastips, mythinspo, mustbethin, edthoughts
B	eatingdisorders, edrecovery, recovery, bodyimage, mentalhealth, recoverywarriors, marchagainsted, edawareness, mentalillness, endstigma, bellletstalk, ended, eds, aedchat, hope, nedawareness, annawestinact, adiosed, endthestigma, rdchat, carers, treatment, eatingdisorder, annaslaw, skinnygirl, selflove, skinnygirlproblems, endthewait

We examine hashtags that each group of users post in the ED-related tweets to study their posting interests. We have shown the most frequent hashtags and their co-occurrence networks used by each group of users in the main text. In order to filter out common terms and obtain a more intuitive comparison, we here use *Normalized Pointwise Mutual Information* (*NPMI*) [12], an information theoretical association measure, to rank the relative prominence of a hashtag in a group of users. Given $f(h, g)$ is the frequency of hashtag h used by users from group $g \in \{A, B\}$, the *NPMI* between h and g is computed as:

$$NPMI(h, g) = \left(\log \frac{P(h, g)}{P(h)P(g)}\right) / \left(-\log P(h, g)\right) = \left(\log \frac{f(h, g)N}{f(h)f(g)}\right) / \left(-\log \frac{f(h, g)}{N}\right) \quad (6)$$

where $f(h) = \sum_g f(h, g)$ is the frequency of a tag used by all users from both two groups, and $f(g) = \sum_h f(h, g)$ is the total frequency of all hashtags used by users in group g. $N = \sum_g \sum_h f(h, g)$ is the total frequency of all tags used by all users. Table S1 shows hashtags that have the largest *NPMI* values in the two groups of users respectively, where *NPMI* is computed only for hashtags that are used in more than three tweets and by more than three distinct users.

3.2 Attitudes on ED-related Content

We categorize the ED-related tweets into "pro-ED", "pro-recovery", "mixed" and "unspecified" themes based on the concurrences of hashtags indicative of a pro-ED and pro-recovery tendency in tweets. Based on previous studies on characterizing pro-ED and pro-recovery content on social media [5, 13, 14, 6, 15, 16], we identify two clusters of hashtags that are indicative of pro-ED and pro-recovery tendencies respectively from the topic clusters of hashtags found in the ED-related tweets (see Fig. S4). After removing generic tags, we obtain 134 pro-ED and 39 pro-recovery tags. Table S2 lists examples of the pro-ED and pro-recovery hashtags we used.

Fig. S5 shows the statistics of users and tweets involved in different themes from the two groups. Table S3 reports the statistics of sentiments (measured by SentiStrength [17]) that the

Table S2: Most frequent pro-ED and pro-recovery hashtags.

Topic	*#Hashtags*
Pro-ED	thinspo, thinspiration, proana, bonespo, proed, legspo, mythinspo, promia, thinspiraton, thinsporation, thinspogoals, thinspos, thinspothursday, thinspo-quotes, proanamia, thinsperation, bonesspo, thinspirationoftheday, thinsp, proanatips, thinspoooo
Pro-recovery	edrecovery, recovery, recoverywarriors, ended, treatment, anorexiarecovery, prorecovery, recoveryispossible, eatingdisorderrecovery, anarecovery, recover, recoverywarrior, edtreatment, recoveryisworthit, teamrecovery, bulimiarecov-ery, recoveryninjas

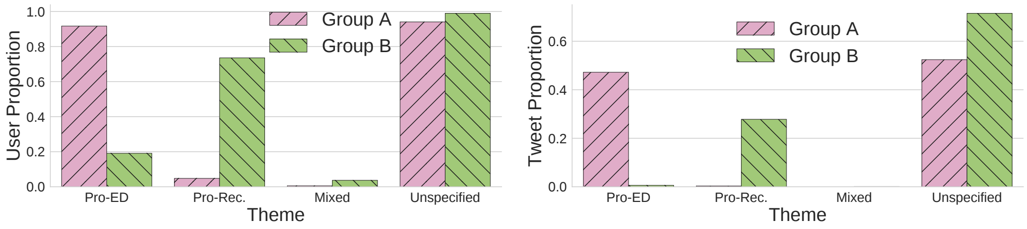

(a) (b)

Figure S5: (a) Proportions of users engaged in different themes from each group. (b) Proportions of tweets involved in different themes posted by each group.

Table S3: Sentiments of two groups of users on different themes of content. "All" denotes all content regardless of their assigned themes. Two-sided Mann–Whitney U tests evaluate the differences of means between groups, significance levels: * $p < 0.05$; ** $p < 0.01$; *** $p < 0.001$.

Theme	*Group A* ($\mu \pm \sigma$)	*Group B* ($\mu \pm \sigma$)	z	p
Pro-ED	0.52 ± 1.20	-0.30 ± 1.28	11.39	0.00 ***
Pro-Rec.	0.14 ± 1.35	0.31 ± 1.25	-3.45	0.00 ***
Mixed	0.23 ± 1.20	0.50 ± 1.36	-0.68	0.50
Unspecified	-0.13 ± 1.39	0.17 ± 1.30	-38.12	0.00 ***
All	0.18 ± 1.34	0.21 ± 1.29	-4.92	0.00 ***

two groups of users express in tweets on different themes. In the main text, we normalize the sentiment scores with z-scores. Given a tweet i posted by user u from group g with a sentiment score $S_{u,i}$, the $z-$score for this sentiment score $z_{u,i}$ is

$$z_{u,i} = \frac{S_{u,i} - \bar{S_g}}{\sigma(S_g)}, \tag{7}$$

where $\bar{S_g}$ and $\sigma(S_g)$ are the mean sentiment of all tweets posted by users from group g and the standard deviation respectively (i.e., items in the "all" line in Table S3).

3.3 Manual Annotation

To verify our results, we go through the Twitter homepages of a random sample of 100 users, where 50 users are from group A and 50 users are from group B. Based on users' posted tweets, images and friends' profiles, we annotate each user into three categories: pro-ED, pro-recovery

and not-sure. We observe that 83 users manifest a pronounced pro-ED or pro-recovery tendency on Twitter, with 39 users from group A and 44 users from group B. If we assume the group A as a pro-ED cohort and the group B as a pro-recovery cohort, the Cohen's κ between our manual annotation and the above clustering analysis on these 83 users is $\kappa = 0.85$.

4 Emotional Interactions

We measure sentiments in inter- and intra-community tweet messages to examine emotional interactions. Based on the community labels of source and target nodes, we categorize interaction links in the communication network into four types: links within the pro-ED community ($L^{ED}_{\circlearrowright}$), links from the pro-ED community to the pro-recovery community ($L^{ED}_{\curvearrowright}$), links within the pro-recovery community ($L^{Rec}_{\circlearrowright}$), and links from the pro-recovery community to the pro-ED community ($L^{Rec}_{\curvearrowright}$). Fig. S6 shows the statistics of users and links on each type of interactions.

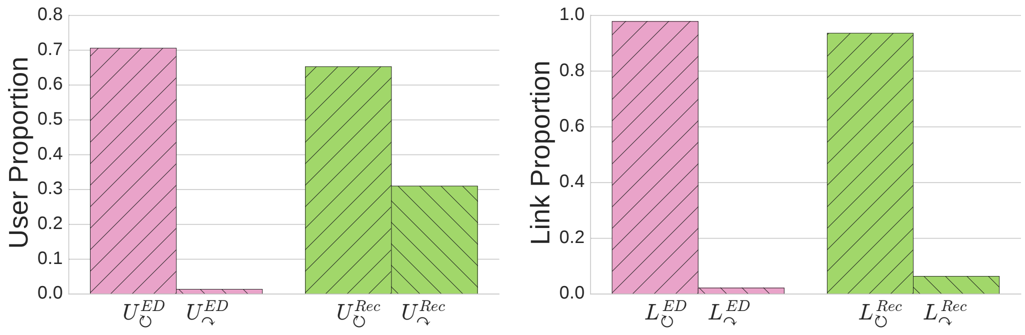

(a) (b)

Figure S6: (a) Proportions of users who launched intra- and inter-community links $U_{\circlearrowright}$ and $U_{\curvearrowright}$ in pro-ED (ED) and pro-recovery (Rec) communities respectively. (b) Proportions of intra- and inter-community links $L_{\circlearrowright}$ and $L_{\curvearrowright}$ over all links sourced from pro-ED (ED) and pro-recovery (Rec) communities respectively. Red and green colours annotate pro-ED and pro-recovery communities repetitively.

Table S4: Means and standard deviations of sentiments in inter- and intra-community messages. Each line describes the statistics of inter- and intra-community interactions sourced from a given community. Two-sided Mann–Whitney U tests evaluate the differences of mean sentiments at each line, significance levels: * $p < 0.05$; ** $p < 0.01$; *** $p < 0.001$.

Community	$\mu_{\circlearrowright} \pm \sigma_{\circlearrowright}$	$\mu_{\curvearrowright} \pm \sigma_{\curvearrowright}$	z	p
Pro-ED	0.44 ± 1.36	0.15 ± 1.33	3.04	0.002 **
Pro-recovery	0.43 ± 1.26	-0.07 ± 1.38	6.82	0.000 ***

Table S4 lists the means and standard deviations of sentiments associated with each type of links. In the main text, we normalize the sentiment scores of links with z-scores. Given a tweet message sent from a user in community i to another user in community j with a sentiment score $S_{i,j}$, the z-score is:

$$z_{i,j} = \frac{S_{i,j} - \bar{S}_i}{\sigma(S_i)}, \quad (8)$$

where $\bar{S}_i$ and $\sigma(S_i)$ are the mean sentiment and standard deviation of all messages sent from users in community i. The mean and standard deviation of sentiments for all messages sent from the

pro-ED community are $\bar{S_{ED}} = 0.43$ and $\sigma(S_{ED}) = 1.36$, while those sent from the pro-recovery community are $\bar{S_{rec}} = 0.40$ and $\sigma(S_{rec}) = 1.27$, significantly different at $p < 0.05$ in a two-sided Mann–Whitney U test.

5 Measures on User Characteristics

We consider 22 measures on social activities and language use in tweets to characterize users' social behaviours and psychometric properties exhibited on Twitter.

5.1 Social Activities

The measures on social activities include:

Social Capital. We measure users' social capital by their overall numbers of followees, tweets and followers observed from their profile information respectively.

Activity. We use the average numbers of followees, tweets and followers per day (from the date of account creation to the date of last post in our observation) to measure the activity of a user.

Interaction Preference. We measure the proportions of tweets that involve different types of interactions (i.e., re-tweeting, mentioning and replying) in a user's most recent tweets we collected. We only consider the mentions that are directly used by a user; any mentions in an original tweet that users re-tweeted are ignored.

Interaction Diversity. We also measure whether a user tends to interact with various individuals or certain specific people. Following previous studies [18, 3], we use entropy as a diversity measure. Given a user u, we track the sequence of people interacted by u (denoted as T_u) in u's historical tweets. The interaction diversity of u in terms of a type of interactions I is measured by the entropy of such interactions with different targets $v \in T_u$:

$$H(u, I) = -\sum_{v \in T_u} p(I_v) \log p(I_v), \tag{9}$$

where $I \in \{\text{re-tweet}, \text{mention}, \text{reply}\}$, and $p(I_v) = \frac{\#I_v}{\sum_{j \in T_u} \#I_j}$. $\#I_v$ is the number of interactions I with target v. Larger entropy values indicate a higher diversity of interests that a user has.

5.2 Language Use

We adopt the psycholinguistic lexicon LIWC [19] to characterize users' language use in tweets. This lexicon decomposes text data into 80 psychologically relevant variables, corresponding to different emotion, linguistic styles, personal concerns, and so on. Based on the cognitive behavioural theory of ED [20], we frame 5 types of variables that measure cognitive attributes and thought patterns associated with ED from LIWC outcomes: (1) concerns of body image, eating behaviours and health, comprising *body* and *ingest* and *health*; (2) interpersonal awareness and focus, comprising *1st personal singular (I)* and *1st personal plural (we)*; (3) social concern, encoded by *social*; (4) abusive language and negation use, measured by *swear* and *negate*; (5) affective processes, comprising positive emotion (*posemo*) and negative emotion (*negemo*).

6 Community Norms

6.1 Characterizing Social Norms

In addition to users' attributes measured by LIWC, we define a metric to measure the tendency that a use promote a pro-ED or pro-recovery tendency (called *pro-strength*). The basic ideal of this metric is that continuously making highly positive comments on pro-ED or pro-recovery content

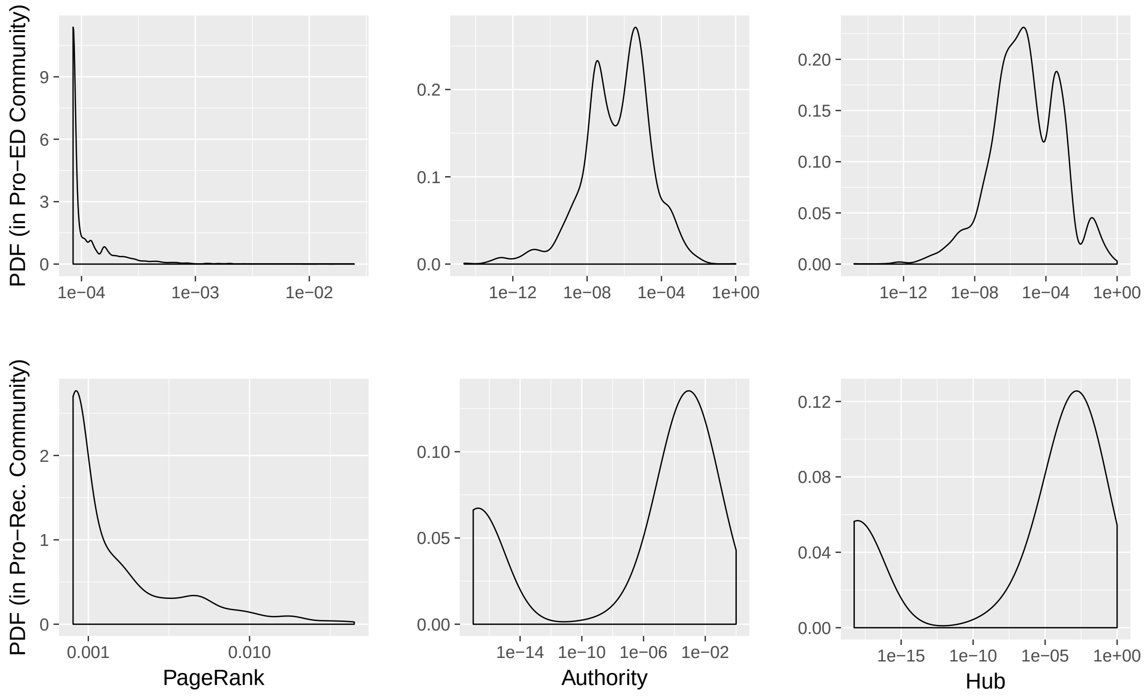

Figure S7: Probability density functions of PageRank, authority and hub centralities measured in pro-ED and pro-recovery communities. The values of centralities are logarithmic scaled (base=10).

indicates a strong tendency of a user to promote a pro-ED or pro-recovery lifestyle and behaviour. Given user u who belongs to a community $c \in \{\text{pro-ED}, \text{pro-recovery}\}$, has totally N_u tweets in our tweet corpus, and posts a set of tweets T_c each of which contains one or more c-related hashtags, the pro-strength of u is:

$$Prostr(u) = \frac{\sum S_u(t|t \in T_c)}{N_u}, \tag{10}$$

where $S_u(t)$ is the sentiment of u in tweet t.

Fig. S7 shows the distributions of PageRank [21], authority and hub centralities (produced by the HITS algorithm [22]) that are measured from the intra-community communication networks among pro-ED and pro-recovery users respectively. Since PageRank gives a constant weight to nodes without any in-degree, the distributions of PageRank centralities are smoother than those of HITS centralities (i.e., without multiple peaks). Note that the ranges of centralities are different across networks with different sizes.

6.2 Regression Models

We use linear robust regression models since these models require less restrictive assumptions, as compared with the least squares regression [23]. Each model predicts the centrality of a user in a communication network based on an attribute of the user (such as concerns on body or positive emotion), along with several covariates that may affect a user's mention and reply interactions on Twitter or the process of measuring network centrality. These covariates include the total numbers of followers (#followees), tweets (#tweets), followers (#followers) that a user has, fractions of historical tweets that the user mentions (%mention) and replies to (%reply) others, and the number of historical tweets that the user has in our data (#historical tweets). Robust regression can be estimated by the iterated re-weighted least squares (IRLS), in which the influence of outliers

(i.e., observations that do not follow the pattern of the other observations) are down-weighted to provide a better fit to the majority of the data. There are several weighting functions that can be used for IRLS. We use the Huber's weighting function [24] in our analysis. The complete lists of variables and their coefficients in each model are reported in Tables S5-S15. Note that we only consider users who are within the giant weakly connected components of the intra-community networks, due to the dominance of the giant components and incomparable PageRank values of nodes across disconnected components. Thus, the numbers of users/observations in the regression analysis are smaller than those reported in the main text.

Table S5: Coefficients estimated for centrality as a function of *body* and covariates. Parentheses refer to standard errors.

	Dependent variable:					
	(PageRank)		(Authority)		(Hub)	
	Pro-ED	Pro-Rec.	Pro-ED	Pro-Rec.	Pro-ED	Pro-Rec.
Body	0.0001**	−0.018*	0.00001***	−0.015	0.00001	−0.019
	(0.00005)	(0.008)	(0.00000)	(0.011)	(0.00003)	(0.032)
#Followees	−0.00001***	−0.0001***	−0.00000***	−0.0001	0.00000**	0.0002
	(0.00000)	(0.00004)	(0.00000)	(0.0001)	(0.00000)	(0.0002)
#Tweets	−0.00000***	0.0002*	−0.00000***	0.0002	0.00000***	−0.0004
	(0.00000)	(0.0001)	(0.00000)	(0.0001)	(0.00000)	(0.0003)
#Followers	0.00002***	0.0002***	0.00000***	0.0002**	−0.00000***	−0.0002
	(0.00000)	(0.00003)	(0.00000)	(0.0001)	(0.00000)	(0.0001)
%Mention	−0.00002***	0.0005*	−0.00000***	0.0003	0.00000	0.004***
	(0.00000)	(0.0002)	(0.00000)	(0.0003)	(0.00000)	(0.001)
%Reply	0.00003***	−0.0001	−0.00000	−0.001*	−0.00000	−0.001
	(0.00000)	(0.0003)	(0.00000)	(0.0005)	(0.00000)	(0.001)
#Historical Tweets	0.000***	−0.00000	0.000***	−0.00000	−0.000	0.00000*
	(0.000)	(0.00000)	(0.000)	(0.00000)	(0.000)	(0.00000)
Constant	0.0001***	−0.0004	0.00000	−0.001	0.00000	0.003
	(0.00000)	(0.0004)	(0.00000)	(0.001)	(0.00000)	(0.002)
Observations	5,584	388	5,584	388	5,584	388
Note:						*p<0.05; **p<0.01; ***p<0.001

References

[1] T. Wang, M. Brede, A. Ianni, and E. Mentzakis, "Detecting and characterizing eating-disorder communities on social media," in *Proceedings of the Tenth International Conference on Web Search and Data Mining (WSDM) 2017*, pp. 91–100, ACM, 2017.

[2] B. D. Davison, "Topical locality in the web," in *Proceedings of the 23rd annual international ACM SIGIR conference on Research and development in information retrieval*, pp. 272–279, ACM, 2000.

Table S6: Coefficients estimated for centrality as a function of *ingest* and covariates. Parentheses refer to standard errors.

	Dependent variable:					
	(PageRank)		(Authority)		(Hub)	
	Pro-ED	Pro-Rec.	Pro-ED	Pro-Rec.	Pro-ED	Pro-Rec.
Ingest	0.0001***	0.006*	0.00001***	0.014***	0.0001***	0.035**
	(0.00003)	(0.003)	(0.00000)	(0.004)	(0.00002)	(0.011)
#Followees	−0.00001***	−0.0001*	−0.00000***	0.00004	0.00000**	0.0003
	(0.00000)	(0.00004)	(0.00000)	(0.0001)	(0.00000)	(0.0002)
#Tweets	−0.00000***	0.0001	−0.00000***	0.0001	0.00000***	−0.0004
	(0.00000)	(0.0001)	(0.00000)	(0.0001)	(0.00000)	(0.0003)
#Followers	0.00002***	0.0002***	0.00000***	0.0001*	−0.00000***	−0.0002
	(0.00000)	(0.00003)	(0.00000)	(0.00005)	(0.00000)	(0.0001)
%Mention	−0.00001***	0.0005*	−0.00000***	0.0005	0.00000	0.004***
	(0.00000)	(0.0002)	(0.00000)	(0.0003)	(0.00000)	(0.001)
%Reply	0.00003***	0.0003	−0.00000	−0.001	−0.00000	0.0004
	(0.00000)	(0.0003)	(0.00000)	(0.0005)	(0.00000)	(0.001)
#Historical Tweets	0.000***	−0.00000	0.000***	0.00000	−0.000	0.00000*
	(0.000)	(0.00000)	(0.000)	(0.00000)	(0.000)	(0.00000)
Constant	0.0001***	−0.001	0.00000	−0.002**	−0.00000	0.001
	(0.00000)	(0.0004)	(0.00000)	(0.001)	(0.00000)	(0.002)
Observations	5,584	388	5,584	388	5,584	388
Note:						*p<0.05; **p<0.01; ***p<0.001

Table S7: Coefficients estimated for centrality as a function of *health* and covariates. Parentheses refer to standard errors.

	Dependent variable:					
	(PageRank)		(Authority)		(Hub)	
	Pro-ED	Pro-Rec.	Pro-ED	Pro-Rec.	Pro-ED	Pro-Rec.
Health	0.0001*	−0.003	0.00000	−0.003	0.00004	0.003
	(0.0001)	(0.004)	(0.00000)	(0.006)	(0.00004)	(0.016)
#Followees	−0.00001***	−0.0001**	−0.00000***	−0.00004	0.00000**	0.0002
	(0.00000)	(0.00004)	(0.00000)	(0.0001)	(0.00000)	(0.0002)
#Tweets	−0.00000***	0.0001*	−0.00000***	0.0002	0.00000***	−0.0004
	(0.00000)	(0.0001)	(0.00000)	(0.0001)	(0.00000)	(0.0003)
#Followers	0.00002***	0.0002***	0.00000***	0.0002**	−0.00000***	−0.0002
	(0.00000)	(0.00003)	(0.00000)	(0.0001)	(0.00000)	(0.0001)
%Mention	−0.00002***	0.0004	−0.00000***	0.0003	0.00000	0.004***
	(0.00000)	(0.0002)	(0.00000)	(0.0003)	(0.00000)	(0.001)
%Reply	0.00003***	−0.00002	−0.00000*	−0.001*	−0.00000	−0.0005
	(0.00000)	(0.0004)	(0.00000)	(0.001)	(0.00000)	(0.001)
#Historical Tweets	0.000***	−0.00000	0.000***	−0.00000	−0.000	0.00000*
	(0.000)	(0.00000)	(0.000)	(0.00000)	(0.000)	(0.00000)
Constant	0.0001***	−0.001	0.00000	−0.001*	0.00000	0.002
	(0.00000)	(0.0004)	(0.00000)	(0.001)	(0.00000)	(0.002)
Observations	5,584	388	5,584	388	5,584	388
Note:						*p<0.05; **p<0.01; ***p<0.001

Table S8: Coefficients estimated for centrality as a function of i and covariates. Parentheses refer to standard errors.

	Dependent variable:					
	(PageRank)		(Authority)		(Hub)	
	Pro-ED	Pro-Rec.	Pro-ED	Pro-Rec.	Pro-ED	Pro-Rec.
I	−0.00004*	−0.005*	−0.00000*	−0.011**	−0.00001	−0.024**
	(0.00002)	(0.002)	(0.00000)	(0.003)	(0.00001)	(0.008)
#Followees	−0.00001***	−0.0001**	−0.00000***	−0.00004	0.00000**	0.0002
	(0.00000)	(0.00004)	(0.00000)	(0.0001)	(0.00000)	(0.0002)
#Tweets	−0.00000***	0.0002*	−0.00000***	0.0003*	0.00000***	−0.0002
	(0.00000)	(0.0001)	(0.00000)	(0.0001)	(0.00000)	(0.0003)
#Followers	0.00002***	0.0002***	0.00000***	0.0001	−0.00000***	−0.0004*
	(0.00000)	(0.00004)	(0.00000)	(0.0001)	(0.00000)	(0.0001)
%Mention	−0.00002***	0.0003	−0.00000***	0.0001	0.00000	0.003***
	(0.00000)	(0.0002)	(0.00000)	(0.0004)	(0.00000)	(0.001)
%Reply	0.00002***	0.0003	−0.00000**	−0.001	−0.00001	0.001
	(0.00000)	(0.0003)	(0.00000)	(0.001)	(0.00000)	(0.001)
#Historical Tweets	0.000**	−0.00000	0.000***	−0.00000	−0.000*	0.00000*
	(0.000)	(0.00000)	(0.000)	(0.00000)	(0.000)	(0.00000)
Constant	0.0001***	−0.0004	0.00000**	−0.001	0.00000	0.003
	(0.00000)	(0.0004)	(0.00000)	(0.001)	(0.00000)	(0.002)
Observations	5,584	388	5,584	388	5,584	388

Note: *p<0.05; **p<0.01; ***p<0.001

Table S9: Coefficients estimated for centrality as a function of *we* and covariates. Parentheses refer to standard errors.

	Dependent variable:					
	(PageRank)		(Authority)		(Hub)	
	Pro-ED	Pro-Rec.	Pro-ED	Pro-Rec.	Pro-ED	Pro-Rec.
We	0.0004*	0.009*	−0.00001	0.007	−0.00001	0.014
	(0.0002)	(0.004)	(0.00001)	(0.006)	(0.0001)	(0.015)
#Followees	−0.00001***	−0.0001**	−0.00000***	−0.00004	0.00000**	0.0002
	(0.00000)	(0.00004)	(0.00000)	(0.0001)	(0.00000)	(0.0002)
#Tweets	−0.00000***	0.0002*	−0.00000***	0.0002	0.00000***	−0.0003
	(0.00000)	(0.0001)	(0.00000)	(0.0001)	(0.00000)	(0.0003)
#Followers	0.00002***	0.0002***	0.00000***	0.0001*	−0.00000***	−0.0003
	(0.00000)	(0.00004)	(0.00000)	(0.0001)	(0.00000)	(0.0001)
%Mention	−0.00002***	0.0004	−0.00000***	0.0003	0.00000	0.003***
	(0.00000)	(0.0002)	(0.00000)	(0.0003)	(0.00000)	(0.001)
%Reply	0.00002***	0.0001	−0.00000*	−0.001*	−0.00001	−0.0005
	(0.00000)	(0.0003)	(0.00000)	(0.0005)	(0.00000)	(0.001)
#Historical Tweets	0.000***	−0.00000	0.000***	−0.000	−0.000	0.00000*
	(0.000)	(0.00000)	(0.000)	(0.00000)	(0.000)	(0.00000)
Constant	0.0001***	−0.001	0.00000*	−0.001*	0.00000	0.002
	(0.00000)	(0.0004)	(0.00000)	(0.001)	(0.00000)	(0.002)
Observations	5,584	388	5,584	388	5,584	388
Note:						*p<0.05; **p<0.01; ***p<0.001

Table S10: Coefficients estimated for centrality as a function of *social* and covariates. Parentheses refer to standard errors.

	Dependent variable:					
	(PageRank)		(Authority)		(Hub)	
	Pro-ED	Pro-Rec.	Pro-ED	Pro-Rec.	Pro-ED	Pro-Rec.
Social	−0.00004*	0.001	−0.00000	0.0004	−0.00000	0.001
	(0.00002)	(0.002)	(0.00000)	(0.002)	(0.00001)	(0.006)
#Followees	−0.00001***	−0.0001**	−0.00000***	−0.00004	0.00000**	0.0002
	(0.00000)	(0.00004)	(0.00000)	(0.0001)	(0.00000)	(0.0002)
#Tweets	−0.00000***	0.0001*	−0.00000***	0.0002	0.00000***	−0.0004
	(0.00000)	(0.0001)	(0.00000)	(0.0001)	(0.00000)	(0.0003)
#Followers	0.00002***	0.0002***	0.00000***	0.0002**	−0.00000***	−0.0002
	(0.00000)	(0.00003)	(0.00000)	(0.0001)	(0.00000)	(0.0001)
%Mention	−0.00002***	0.0004	−0.00000***	0.0003	0.00000	0.004***
	(0.00000)	(0.0002)	(0.00000)	(0.0003)	(0.00000)	(0.001)
%Reply	0.00003***	0.0001	−0.00000	−0.001*	−0.00001	−0.001
	(0.00000)	(0.0003)	(0.00000)	(0.001)	(0.00000)	(0.001)
#Historical Tweets	0.000***	−0.00000	0.000***	−0.000	−0.000	0.00000*
	(0.000)	(0.00000)	(0.000)	(0.00000)	(0.000)	(0.00000)
Constant	0.0001***	−0.001	0.00000*	−0.001*	0.00000	0.002
	(0.00000)	(0.0004)	(0.00000)	(0.001)	(0.00000)	(0.002)
Observations	5,584	388	5,584	388	5,584	388
Note:						*p<0.05; **p<0.01; ***p<0.001

Table S11: Coefficients estimated for centrality as a function of *swear* and covariates. Parentheses refer to standard errors.

	Dependent variable:					
	(PageRank)		(Authority)		(Hub)	
	Pro-ED	Pro-Rec.	Pro-ED	Pro-Rec.	Pro-ED	Pro-Rec.
Swear	−0.0002*	−0.009	−0.00000	−0.048	−0.0001	−0.241**
	(0.0001)	(0.022)	(0.00000)	(0.033)	(0.00004)	(0.090)
#Followees	−0.00001***	−0.0001**	−0.00000***	−0.00004	0.00000**	0.0002
	(0.00000)	(0.00004)	(0.00000)	(0.0001)	(0.00000)	(0.0002)
#Tweets	−0.00000***	0.0001*	−0.00000***	0.0002	0.00000***	−0.0003
	(0.00000)	(0.0001)	(0.00000)	(0.0001)	(0.00000)	(0.0003)
#Followers	0.00002***	0.0002***	0.00000***	0.0001*	−0.00000***	−0.0003*
	(0.00000)	(0.00004)	(0.00000)	(0.0001)	(0.00000)	(0.0001)
%Mention	−0.00002***	0.0004	−0.00000***	0.0003	0.00000	0.003***
	(0.00000)	(0.0002)	(0.00000)	(0.0003)	(0.00000)	(0.001)
%Reply	0.00002***	0.0001	−0.00000*	−0.001*	−0.00001*	−0.0003
	(0.00000)	(0.0003)	(0.00000)	(0.0005)	(0.00000)	(0.001)
#Historical Tweets	0.000***	−0.00000	0.000***	0.000	−0.000	0.00000*
	(0.000)	(0.00000)	(0.000)	(0.00000)	(0.000)	(0.00000)
Constant	0.0001***	−0.001	0.00000*	−0.001	0.00000	0.003
	(0.00000)	(0.0004)	(0.00000)	(0.001)	(0.00000)	(0.002)
Observations	5,584	388	5,584	388	5,584	388
Note:						*p<0.05; **p<0.01; ***p<0.001

Table S12: Coefficients estimated for centrality as a function of *negate* and covariates. Parentheses refer to standard errors.

	Dependent variable:					
	(PageRank)		(Authority)		(Hub)	
	Pro-ED	Pro-Rec.	Pro-ED	Pro-Rec.	Pro-ED	Pro-Rec.
Negate	−0.0001*	−0.011	−0.00001**	−0.017	−0.00003	−0.044
	(0.0001)	(0.006)	(0.00000)	(0.010)	(0.00003)	(0.025)
#Followees	−0.00001***	−0.0001**	−0.00000***	−0.00004	0.00000**	0.0002
	(0.00000)	(0.00004)	(0.00000)	(0.0001)	(0.00000)	(0.0002)
#Tweets	−0.00000***	0.0002*	−0.00000***	0.0002	0.00000***	−0.0003
	(0.00000)	(0.0001)	(0.00000)	(0.0001)	(0.00000)	(0.0003)
#Followers	0.00002***	0.0002***	0.00000***	0.0001*	−0.00000***	−0.0003*
	(0.00000)	(0.00004)	(0.00000)	(0.0001)	(0.00000)	(0.0001)
%Mention	−0.00002***	0.0004	−0.00000***	0.0002	0.00000	0.003***
	(0.00000)	(0.0002)	(0.00000)	(0.0004)	(0.00000)	(0.001)
%Reply	0.00002***	0.0001	−0.00000**	−0.001*	−0.00001	−0.0004
	(0.00000)	(0.0003)	(0.00000)	(0.001)	(0.00000)	(0.001)
#Historical Tweets	0.000***	−0.00000	0.000***	−0.00000	−0.000*	0.00000*
	(0.000)	(0.00000)	(0.000)	(0.00000)	(0.000)	(0.00000)
Constant	0.0001***	−0.0004	0.00000**	−0.001	0.00000	0.003*
	(0.00000)	(0.0004)	(0.00000)	(0.001)	(0.00000)	(0.002)
Observations	5,584	388	5,584	388	5,584	388
Note:						*p<0.05; **p<0.01; ***p<0.001

Table S13: Coefficients estimated for centrality as a function of *posemo* and covariates. Parentheses refer to standard errors.

	Dependent variable:					
	(PageRank)		(Authority)		(Hub)	
	Pro-ED	Pro-Rec.	Pro-ED	Pro-Rec.	Pro-ED	Pro-Rec.
Posemo	−0.00002	−0.004	0.00000	−0.005	−0.00003	0.0004
	(0.00003)	(0.003)	(0.00000)	(0.004)	(0.00002)	(0.011)
#Followees	−0.00001***	−0.0001**	−0.00000***	−0.00003	0.00000**	0.0002
	(0.00000)	(0.00004)	(0.00000)	(0.0001)	(0.00000)	(0.0002)
#Tweets	−0.00000***	0.0002*	−0.00000***	0.0002	0.00000***	−0.0004
	(0.00000)	(0.0001)	(0.00000)	(0.0001)	(0.00000)	(0.0003)
#Followers	0.00002***	0.0002***	0.00000***	0.0002**	−0.00000***	−0.0002
	(0.00000)	(0.00004)	(0.00000)	(0.0001)	(0.00000)	(0.0001)
%Mention	−0.00002***	0.001*	−0.00000***	0.0004	0.00000	0.003***
	(0.00000)	(0.0002)	(0.00000)	(0.0003)	(0.00000)	(0.001)
%Reply	0.00003***	0.0002	−0.00000**	−0.001	−0.00000	−0.001
	(0.00001)	(0.0004)	(0.00000)	(0.001)	(0.00000)	(0.001)
#Historical Tweets	0.000***	−0.00000	0.000***	−0.00000	−0.000	0.00000*
	(0.000)	(0.00000)	(0.000)	(0.00000)	(0.000)	(0.00000)
Constant	0.0001***	−0.001	0.00000	−0.001	0.00000	0.002
	(0.00000)	(0.0004)	(0.00000)	(0.001)	(0.00000)	(0.002)
Observations	5,584	388	5,584	388	5,584	388
Note:						*p<0.05; **p<0.01; ***p<0.001

Table S14: Coefficients estimated for centrality as a function of *negemo* and covariates. Parentheses refer to standard errors.

	Dependent variable:					
	(PageRank)		(Authority)		(Hub)	
	Pro-ED	Pro-Rec.	Pro-ED	Pro-Rec.	Pro-ED	Pro-Rec.
Negemo	−0.0001**	−0.013**	−0.00001***	−0.018*	−0.0001***	−0.066***
	(0.00003)	(0.005)	(0.00000)	(0.008)	(0.00002)	(0.020)
#Followees	−0.00001***	−0.0001**	−0.00000***	−0.00005	0.00000**	0.0002
	(0.00000)	(0.00004)	(0.00000)	(0.0001)	(0.00000)	(0.0002)
#Tweets	−0.00000***	0.0001*	−0.00000***	0.0002	0.00000***	−0.0004
	(0.00000)	(0.0001)	(0.00000)	(0.0001)	(0.00000)	(0.0003)
#Followers	0.00002***	0.0002***	0.00000***	0.0001*	−0.00000***	−0.0003*
	(0.00000)	(0.00003)	(0.00000)	(0.0001)	(0.00000)	(0.0001)
%Mention	−0.00002***	0.0003	−0.00000***	0.0002	0.00000	0.003***
	(0.00000)	(0.0002)	(0.00000)	(0.0004)	(0.00000)	(0.001)
%Reply	0.00002***	0.00001	−0.00000***	−0.001*	−0.00001**	−0.001
	(0.00000)	(0.0003)	(0.00000)	(0.001)	(0.00000)	(0.001)
#Historical Tweets	0.000**	−0.00000	0.000***	0.00000	−0.000*	0.00000*
	(0.000)	(0.00000)	(0.000)	(0.00000)	(0.000)	(0.00000)
Constant	0.0001***	−0.0001	0.00000**	−0.001	0.00000	0.005*
	(0.00000)	(0.0004)	(0.00000)	(0.001)	(0.00000)	(0.002)
Observations	5,584	388	5,584	388	5,584	388
Note:						*p<0.05; **p<0.01; ***p<0.001

Table S15: Coefficients estimated for centrality as a function of *prostrr* and covariates. Parentheses refer to standard errors.

	Dependent variable:					
	(PageRank)		(Authority)		(Hub)	
	Pro-ED	Pro-Rec.	Pro-ED	Pro-Rec.	Pro-ED	Pro-Rec.
Prostrr	0.0001***	0.004*	0.00001***	0.004	−0.00000	0.039***
	(0.00001)	(0.002)	(0.00000)	(0.002)	(0.00001)	(0.006)
#Followees	−0.00001***	−0.0001***	−0.00000***	−0.0001	0.00000**	0.0001
	(0.00000)	(0.00004)	(0.00000)	(0.0001)	(0.00000)	(0.0002)
#Tweets	−0.00000***	0.0002*	−0.00000***	0.0002	0.00000***	−0.0003
	(0.00000)	(0.0001)	(0.00000)	(0.0001)	(0.00000)	(0.0003)
#Followers	0.00002***	0.0003***	0.00000***	0.0002**	−0.00000***	−0.0002
	(0.00000)	(0.00004)	(0.00000)	(0.0001)	(0.00000)	(0.0001)
%Mention	−0.00002***	0.0005*	−0.00000***	0.0004	0.00000	0.004***
	(0.00000)	(0.0002)	(0.00000)	(0.0003)	(0.00000)	(0.001)
%Reply	0.00003***	0.0001	−0.00000*	−0.001*	−0.00001	−0.0003
	(0.00000)	(0.0003)	(0.00000)	(0.001)	(0.00000)	(0.001)
#Historical Tweets	0.000**	−0.00000	0.000***	−0.00000	−0.000	0.00000*
	(0.000)	(0.00000)	(0.000)	(0.00000)	(0.000)	(0.00000)
Constant	0.0001***	−0.001	0.00000	−0.002*	0.00000	0.002
	(0.00000)	(0.0004)	(0.00000)	(0.001)	(0.00000)	(0.002)
Observations	5,584	388	5,584	388	5,584	388
Note:						*p<0.05; **p<0.01; ***p<0.001

[3] L. Weng and F. Menczer, "Topicality and impact in social media: diverse messages, focused messengers," *PloS one*, vol. 10, no. 2, p. e0118410, 2015.

[4] M. Rosvall and C. T. Bergstrom, "Maps of random walks on complex networks reveal community structure," *Proceedings of the National Academy of Sciences*, vol. 105, no. 4, pp. 1118–1123, 2008.

[5] S. Chancellor, T. Mitra, and M. De Choudhury, "Recovery amid pro-anorexia: Analysis of recovery in social media," in *Proceedings of the 2016 CHI Conference on Human Factors in Computing Systems*, pp. 2111–2123, ACM, 2016.

[6] M. De Choudhury, "Anorexia on tumblr: A characterization study," in *Proceedings of the 5th International Conference on Digital Health 2015*, pp. 43–50, ACM, 2015.

[7] A. S. Juarascio, A. Shoaib, and C. A. Timko, "Pro-eating disorder communities on social networking sites: a content analysis," *Eating disorders*, vol. 18, no. 5, pp. 393–407, 2010.

[8] B. Gonçalves, N. Perra, and A. Vespignani, "Modeling users' activity on twitter networks: Validation of dunbar's number," *PloS one*, vol. 6, no. 8, p. e22656, 2011.

[9] B. A. Huberman, D. M. Romero, and F. Wu, "Social networks that matter: Twitter under the microscope," 2008.

[10] P. J. Rousseeuw, "Silhouettes: a graphical aid to the interpretation and validation of cluster analysis," *Journal of computational and applied mathematics*, vol. 20, pp. 53–65, 1987.

[11] L. Kaufman and P. J. Rousseeuw, *Finding groups in data: an introduction to cluster analysis*, vol. 344. John Wiley & Sons, 2009.

[12] G. Bouma, "Normalized (pointwise) mutual information in collocation extraction," in *Proceedings of the Biennial GSCL Conference*, vol. 156, 2009.

[13] A. Arseniev-Koehler, H. Lee, T. McCormick, and M. A. Moreno, "# proana: Pro-eating disorder socialization on twitter," *Journal of Adolescent Health*, vol. 58, no. 6, pp. 659–664, 2016.

[14] A. Oksanen, D. Garcia, A. Sirola, M. Näsi, M. Kaakinen, T. Keipi, and P. Räsänen, "Pro-anorexia and anti-pro-anorexia videos on youtube: Sentiment analysis of user responses," *Journal of medical Internet research*, vol. 17, no. 11, 2015.

[15] S. Syed-Abdul, L. Fernandez-Luque, W.-S. Jian, Y.-C. Li, S. Crain, M.-H. Hsu, Y.-C. Wang, D. Khandregzen, E. Chuluunbaatar, P. A. Nguyen, *et al.*, "Misleading health-related information promoted through video-based social media: anorexia on youtube," *Journal of medical Internet research*, vol. 15, no. 2, p. e30, 2013.

[16] E. Yom-Tov, L. Fernandez-Luque, I. Weber, and S. P. Crain, "Pro-anorexia and pro-recovery photo sharing: a tale of two warring tribes," *Journal of medical Internet research*, vol. 14, no. 6, p. e151, 2012.

[17] M. Thelwall, K. Buckley, G. Paltoglou, D. Cai, and A. Kappas, "Sentiment strength detection in short informal text," *Journal of the Association for Information Science and Technology*, vol. 61, no. 12, pp. 2544–2558, 2010.

[18] N. Eagle, M. Macy, and R. Claxton, "Network diversity and economic development," *Science*, vol. 328, no. 5981, pp. 1029–1031, 2010.

[19] Y. R. Tausczik and J. W. Pennebaker, "The psychological meaning of words: Liwc and computerized text analysis methods," *Journal of language and social psychology*, vol. 29, no. 1, pp. 24–54, 2010.

[20] C. G. Fairburn, R. Shafran, and Z. Cooper, "A cognitive behavioural theory of anorexia nervosa," *Behaviour Research and Therapy*, vol. 37, no. 1, pp. 1–13, 1999.

[21] L. Page, S. Brin, R. Motwani, and T. Winograd, "The pagerank citation ranking: Bringing order to the web.," tech. rep., Stanford InfoLab, 1999.

[22] J. M. Kleinberg, "Authoritative sources in a hyperlinked environment," *Journal of the ACM (JACM)*, vol. 46, no. 5, pp. 604–632, 1999.

[23] R. Andersen, *Modern methods for robust regression*. No. 152, Sage, 2008.

[24] P. J. Huber *et al.*, "Robust estimation of a location parameter," *The Annals of Mathematical Statistics*, vol. 35, no. 1, pp. 73–101, 1964.
